# Self-renewal of CD133^hi^ cells by IL6/Notch3 signalling regulates endocrine resistance in metastatic breast cancer

**DOI:** 10.1038/ncomms10442

**Published:** 2016-02-09

**Authors:** Pasquale Sansone, Claudio Ceccarelli, Marjan Berishaj, Qing Chang, Vinagolu K. Rajasekhar, Fabiana Perna, Robert L. Bowman, Michele Vidone, Laura Daly, Jennifer Nnoli, Donatella Santini, Mario Taffurelli, Natalie N. C. Shih, Michael Feldman, Jun J. Mao, Christopher Colameco, Jinbo Chen, Angela DeMichele, Nicola Fabbri, John H. Healey, Monica Cricca, Giuseppe Gasparre, David Lyden, Massimiliano Bonafé, Jacqueline Bromberg

**Affiliations:** 1 Department of Medicine, Memorial Sloan Kettering Cancer Center, New York, New York 10021, USA; 2 Department of Experimental, Diagnostic and Specialty Medicine, AlmaMater Studiorum, Universita' di Bologna, Bologna 40138, Italy; 3 Molecular Pharmacology and Chemistry Program, Memorial Sloan Kettering Cancer Center, New York, New York 10021, USA; 4 Cancer Biology and Genetics Program, Memorial Sloan Kettering Cancer Center, New York, New York 10021, USA; 5 Department of Medical and Surgical Sciences, AlmaMater Studiorum, Universita' di Bologna, Bologna 40138, Italy; 6 Pathology Unit, Policlinico S.Orsola-Malpighi University Hospital, Bologna 40138, Italy; 7 Department of Pathology and Laboratory Medicine, Hospital of the University of Pennsylvania, Philadelphia, Pennsylvania 19104, USA; 8 Department of Biostatistics and Epidemiology, Abramson Cancer Center, University of Pennsylvania, Philadelphia, Pennsylvania 19104, USA; 9 Department of Medicine, Abramson Cancer Center, University of Pennsylvania, Philadelphia, Pennsylvania 19104, USA; 10 Orthopedics Service, Memorial Sloan Kettering Cancer Center, New York, New York 10021, USA; 11 Department of Pediatrics, Memorial Sloan Kettering Cancer Center, New York, New York 10021, USA; 12 Department of Pediatrics, Cell and Developmental Biology, Children's Cancer and Blood Foundation Laboratories, Weill Cornell Medical College, New York, New York 10021, USA; 13 CRBA Laboratory, Policlinico Universitario S. Orsola-Malpighi, Bologna 40138, Italy; 14 Department of Medicine, Weill Cornell Medical College, New York, New York 10021, USA

## Abstract

The mechanisms of metastatic progression from hormonal therapy (HT) are largely unknown in luminal breast cancer. Here we demonstrate the enrichment of CD133^hi^/ER^lo^ cancer cells in clinical specimens following neoadjuvant endocrine therapy and in HT refractory metastatic disease. We develop experimental models of metastatic luminal breast cancer and demonstrate that HT can promote the generation of HT-resistant, self-renewing CD133^hi^/ER^lo^/IL6^hi^ cancer stem cells (CSCs). HT initially abrogates oxidative phosphorylation (OXPHOS) generating self-renewal-deficient cancer cells, CD133^hi^/ER^lo^/OXPHOS^lo^. These cells exit metabolic dormancy via an IL6-driven feed-forward ER^lo^-IL6^hi^-Notch^hi^ loop, activating OXPHOS, in the absence of ER activity. The inhibition of IL6R/IL6-Notch pathways switches the self-renewal of CD133^hi^ CSCs, from an IL6/Notch-dependent one to an ER-dependent one, through the re-expression of ER. Thus, HT induces an OXPHOS metabolic editing of luminal breast cancers, paradoxically establishing HT-driven self-renewal of dormant CD133^hi^/ER^lo^ cells mediating metastatic progression, which is sensitive to dual targeted therapy.

## Abstract (web summary)

ER^+^ breast cancer patients treated with endocrine therapies often acquire resistance and develop metastasis. In this study, the authors demonstrate that endocrine therapies can promote the self-renewal of CD133^hi^/ER^lo^ drug resistant cells with metastatic potential driven through the IL6-Notch3 axis activation.

## Graphical abstract

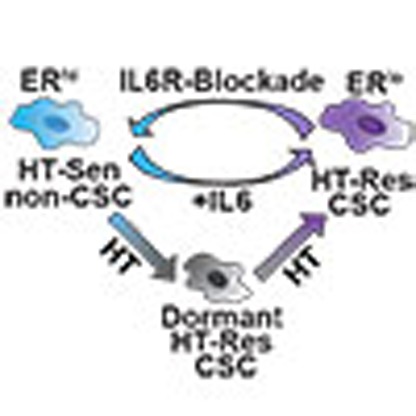

Canonical cancer stem cell (CSC) phenotypes—CD44^hi^/CD24^lo^cells and ALDH^hi^—have been documented to sustain tumour growth and resistance to conventional anticancer therapies (for example, anti-Her2 and chemotherapy/radiation therapy) in several tumour models^1^,^2^,^3^. However, discrepancies in CSC phenotypes and abundance are quite variable in clinical specimens, suggesting that CSCs likely evolve with primary tumour growth, with metastatic progression and in response to therapies^4^,^5^. Indeed, the acquisition of novel genetic changes, including gain of function mutations in the *ESR1* gene, loss of PTEN and discordant expression of Her2 protein, has been observed in ∼20% of metastases following conventional anticancer therapies^6^,^7^,^8^. In addition, a reduction in oestrogen receptor alpha (ER) expression as well as a discrepancy in ER levels between primary tumours and metastatic disease are often observed with the development of tamoxifen resistance without changes in Her2 expression (∼80% of cases)^9^,^10^,^11^.

Although decreased expression of ER, increased circulating interleukin 6 (IL6) levels and the presence of circulating CSCs have independently been associated with metastatic progression in breast cancer patients^11^,^12^,^13^, no models have been proposed to explain their role in endocrine-resistant disease. In this manuscript, we developed the hypothesis that resistance to hormonal therapy (HT) occurs through a change in the self-renewal capacity of metastases, evolving from an ER-dependent to an ER-independent one. We generated experimental and patient-derived models of HT-resistant metastases and determined the evolution of a feed-forward ER-CD133-IL6R-IL6-Notch loop underlying the process of HT resistance. These observations led to therapeutic interventions reversing HT-resistant diseases.

## Results

### Increased CD133 and IL6 expression in HT-resistant cancers

**Figure 1:**
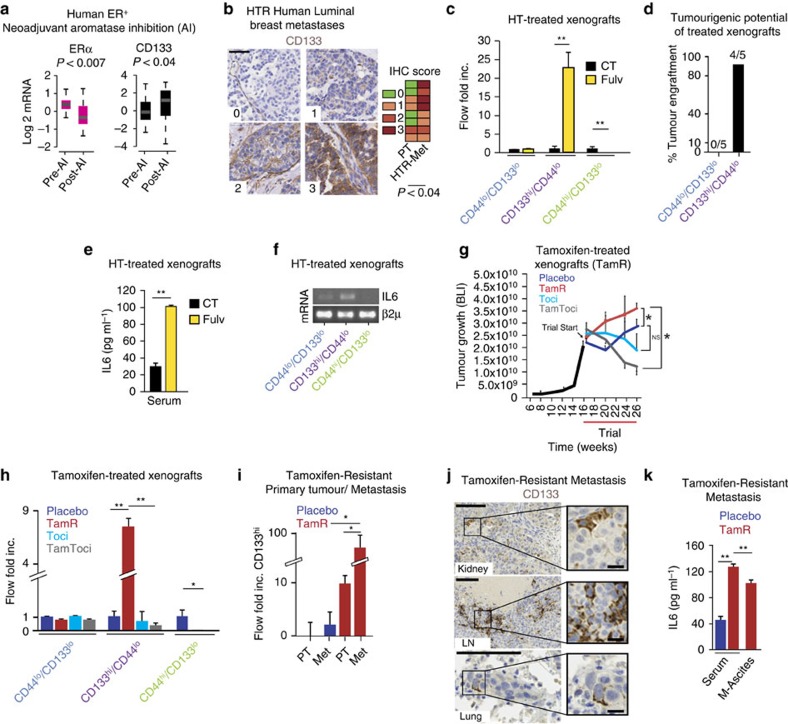
Increased CD133 and IL6 expression in HT-resistant cancers. (**a**) Box plot of CD133 and ERα mRNA levels (GSE1028) in breast cancer tissues pre- and post-Letrozole neoadjuvant treatment. *P* refers to Wald's test; each value corresponds to a patient sample (median, maximum and minimum values are reported). (**b**) CD133 expression [immunohistochemical (IHC) scores 0–3] in matched primary and metastatic tissues from patients who developed HTR metastasis (Supplementary Table 1). Representative images are shown (scale bar, 50 μM). (**c**) CD133 and CD44 cells from vehicle (CT) and fulvestrant (Fulv)-resistant MCF7 xenografts were quantified using flow analysis (Flow Fold Increase, *n*=4 per group, see Methods). (**d**) Bar graph of percentage of mice developing tumours (*n*=5 mice per group, tumour engraftment) from the injection of 100 Fulv-resistant ZR751-derived CD133^hi^/CD44^lo^ or CD133^lo^/CD44^lo^ cancer cells (*P*<0.001, Monte Carlo *χ*^2^-test). (**e**) IL6 levels were measured by ELISA from the serum of HT-resistant tumour-bearing mice (Fulv-resistant MCF7 tumours). (**f**) IL6 RT–PCR analysis from FACS-sorted populations derived from TamR-MCF7 tumours. Beta2 microglobulin (β2μ) mRNA is reported as quantitative control. (**g**) Representative growth kinetics (by bioluminescence (BLI)) of TamR-MCF7 tumours. MCF7 (GFP/Luciferase+) xenografts were established and mice were treated (*n*=5 per group) with tamoxifen citrate pellets (Tam, 5 mg per pellet) with/without tocilizumab (Toci) and in combination, TamToci. The mean BLI value±s.e.m. is reported for each time point. (**h**) MCF7 tumours from **g** were analysed for the expression of CD133 and CD44 using flow analysis (Flow Fold Inc is fold-increased as compared with placebo). (**i**) Flow Fold Increase (log-scale) of CD133^hi^ cells derived from primary tumours and matched metastasis from TamR versus placebo mice. (**j**) Representative IHC images of CD133 protein expression in metastatic tissues (kidney, lymph node, lung) from TamR mice. Scale bar, 100 μm (inserts, 20 μm). (**k**) ELISA of circulating IL6 from TamR and placebo mice (malignant ascites, M-Ascites). Data are reported as mean±s.d. of three independent experiments (*n*=3, **c**,**e**,**h**,**i**,**k**). *P* values (*P<0.05, **P<0.0001) refer to Wilcoxon exact test (**b**), *t*-test (**c**,**e**), multiple comparisons corrected *post hoc t*-test after GLM analysis of variance (ANOVA; **h**,**i**,**k**), repeated measures GLM (**g**).

We hypothesized that HT and resistance to HT would lead to the expansion of cells expressing the CSC marker CD133 in patients with ER+ breast cancer. Luminal (ER+) breast tumours were sampled before and after neoadjuvant HT; specifically aromatase inhibition (letrozole) and the expression of CD133 mRNA (a marker for CSCs) increased (*P*<0.04; Wald's test) as ER mRNA levels decreased (*P*<0.007; Wald's test) in the post-aromatase inhibition tumour samples (Fig. 1a, GSE10281)^14^. In addition, we determined that CD133 expression was elevated in metastatic tissues of patients who developed resistance to HT as compared with their matched primary tumours using immunohistochemical analysis (Fig. 1b, HTR-Met, *P*=0.031; Wilcoxon exact test and Supplementary Table 1).

To corroborate these findings, we developed *in vivo* models of HT-resistant (HTR) disease (MCF7, ZR75 xenografts with tumorigenic capacity were established in the absence of oestradiol, see Methods) and treated with fulvestrant or vehicle for 2 months. Increased levels of CD133^hi^ cells were identified in tumours from HT (fulvestrant)-treated tumour-bearing mice compared with vehicle control (Fig. 1c and Supplementary Fig. 1a). Notably, cells expressing CD44 (another stem cell marker) were not enriched in response to HT in these models. Similarly, HT treatment of tumour-derived cells led to the generation of CD133^hi^ cells *in vitro*, which had potent tumorigenic potential in contrast to the HT-naive (CD133^lo^/CD44^lo^) population (Fig. 1d and Supplementary Fig. 1b,c). Xenograft-derived CD133^hi^/CD44^lo^ cells had increased expression of pluripotent stem cell pathway genes^15^,^16^ as compared with CD44^hi^/CD133^lo^ cells by gene set enrichment microarray mRNA analysis (Supplementary Fig. 1d and Supplementary Data 1). Moreover, CD133^hi^/CD44^lo^ cells had reduced levels of CD24, ALDH1 and CD44 and increased expression of the HTR/CSC marker ABCG2 (ref. 17) as compared with CD44^hi^/CD133^lo^ cells (Supplementary Fig. 1e and Supplementary Data 1, GSE69280). In agreement with previous investigations^1^, CD44^hi^/CD133^lo^ cells express low levels of CD24 compared with the non-stem cell population CD133^lo^/CD44^lo^ (Supplementary Data 1). Thus, both CD133^hi^/CD44^lo^ and CD44^hi^/CD133^lo^ had tumorigenic potential on mammary fat pad (MFP) re-engraftment, as few as 50 orthotopically implanted CSCs, and were able to generate a tumour (Supplementary Fig. 1f) and could form tertiary mammosphere (MS) *in vitro* (Supplementary Fig. 1g). Although both CD44^hi^ and CD133^hi^ cells display CSC features, CD133^hi^ CSCs are preferentially enriched following HT and promote the self-renewal of luminal metastases after the suppression of oestrogen receptor activity.

Because ER is a known repressor of IL6 gene expression^18^, we determined whether HT-treated cells/tumour-bearing mice would lead to increased IL6 expression. Accordingly, secreted IL6 and IL6 promoter activity was elevated in cultured cells derived from tumours and metastases as well as in the serum of mice bearing HT-resistant xenografts (treatment with tamoxifen or fulvestrant; Fig. 1e and Supplementary Fig. 1h,i). Importantly, IL6 mRNA expression was preferentially increased in xenograft-derived CD133^hi^ cells (Fig. 1f), and the self-renewal potential (secondary MS formation) of these cells was further enhanced with exogenous IL6 (Supplementary Fig. 1j,k).

### IL6R blockade re-sensitizes HTR metastasis to HT

These findings led us to examine the consequences of perturbing ER/IL6 signalling on tumour growth and the development of HT resistance. Mice bearing established MCF7 xenografts were treated with HT (tamoxifen) and an IL6R-blocking antibody (tocilizumab), alone or in combination. In these experiments, single therapy alone (tamoxifen or tocilizumab) did not exert significant antitumorigenic effects in the preclinical xenograft trials. Tamoxifen either promoted (tamoxifen-resistant, TamR) or led to a partial reduction in tumour growth (tamoxifen partial resistant, TamR2) compared with controls, while the tamoxifen/tocilizumab regimen potently reduced tumour burden and/or prevented growth in the TamR tumours (Fig. 1g and Supplementary Fig. 2a). Highly resistant TamR tumours were enriched for CD133^hi^ and were deficient for CD44^hi^ CSCs (Supplementary Fig. 2b). Compared with control and tocilizumab-treated tumours, TamR tumours not only had higher numbers of CD133^hi^ cells but also had reduced ER expression, increased circulating IL6 levels and elevated Her2 expression (Fig. 1h and Supplementary Fig. 2c–e). Importantly, the addition of IL6R-blocking antibody (tocilizumab) reversed all of these tamoxifen-induced phenotypes (Fig. 1h and Supplementary Fig. 2c–e). In agreement with these observations, IL6 treatment of ER expressing breast cancer cells (MCF7, T47D) led to a reduction in ER protein expression, ER promoter-driven luciferase and oestrogen response element (ERE)-dependent transcription, which were reversed with tocilizumab (Supplementary Fig. 2f,g). Overall, these data demonstrate fully tamoxifen-resistant tumours (TamR) are enriched with CD133^hi^ cells, which develop in an IL6–IL6R–ER-dependent manner. Conversely, tumours with a partial response to tamoxifen developed fewer CD133^hi^ cells and did not benefit as markedly from the combo treatment (tamoxifen+tocilizumab).

To determine the ER/CD133/IL6 interplay in the development of HT-resistant metastatic disease, we generated an *in vivo* model of metastatic progression. Briefly, tumour-bearing mice were treated with HT and, when primary tumours were ∼1 cm, they were removed and the mice continued to receive HT (tamoxifen or fulvestrant), and metastatic burden was followed by *in vivo* imaging (IVIS luciferase) and confirmed at necropsy. Although the tamoxifen treatment reduced the incidence of metastatic disease in tumour-bearing mice, 5% of the animals developed widely metastatic disease, recapitulating the canonical metastatic spread observed in humans (Supplementary Fig. 3a,b, TamR—lymph nodes, peritoneum and bones, Supplementary Table 2). Interestingly, TamR metastases were markedly enriched (∼30-fold) for CD133^hi^ cells and had an increased expression of secreted/autocrine IL6 compared with the primary tumours (from the same mouse) or from both primary tumours and metastases from placebo-treated mice (Fig. 1i–k and Supplementary Fig. 3c).

**Figure 2:**
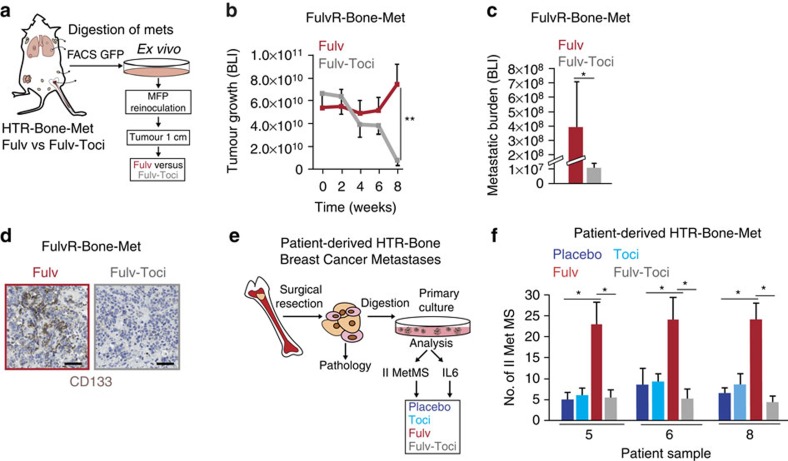
IL6R blockade re-sensitizes HTR metastasis to HT and reduces CD133. (**a**) Schematic of the experimental plan: (1) metastatic cells were isolated via FACS (GFP+) from mice with bone metastases resistant to Fulv (MCF7 derived-FulvR-B-Met, Supplementary Table 2); (2) cells were propagated *ex vivo* for 2 weeks and re-inoculated in the MFP of a new cohort of mice; (3) when tumours reached ∼1 cm diameter, mice were randomized to receive Fulv in the presence/absence of tocilizumab. (**b**,**c**) Tumour/metastatic growth/burden assessed using BLI of FulvR-Bone-Met-bearing mice treated with Fulv and Fulv-Toci. The mean BLI value±s.e.m. is reported for each time point (*n*=5 mice per group). (**d**) Representative CD133 IHC images and histological examination of tumour tissues derived from **b** (scale bar, 50 μm). (**e**) Schematic of the experimental design for the generation of patient-derived bone metastatic cultures (HTR-Bone-Met, Supplementary Table 3). (**f**) Secondary MS growth (Number-No-of II MetMS >100 μm) of patient-derived HTR-Bone-Mets (samples 5, 6 and 8) treated with vehicle (Placebo), Fulv (10 μM, 7 days), tocilizumab (Toci, 50 μg ml^−1^, 7 days) and Fulv-Toci (7 days). Data are reported as mean±s.d. of three independent experiments (*n*=3; **f**). *P* values (*P<0.05, **P<0.0001) refer to *t*-test (**c**), *t*-test after GLM ANOVA (**f**) and *post hoc t*-test corrected for multiple comparisons after GLM for repeated measures (**b**).

To determine whether the ER-IL6 feedback loop promoted the metastatic potential of HT-resistant disease, we first isolated tamoxifen- and fulvestrant-resistant (TamR/FulvR) metastatic lesions from different organs (*n*=13, including bones and lymph nodes). Notably, these metastases were genotypically identical to their matched primary tumours (Supplementary Table 2). To determine whether IL6 blockade (tocilizumab) would influence sensitivity to HT, FulvR bone metastases (FulvR-Bone-Met) were isolated, passaged and re-injected into new cohorts of mice: once tumours reached ∼1 cm, mice were randomized to receive fulvestrant with or without tocilizumab (Fig. 2a: Fulv, Fulv-Toci). The addition of tocilizumab to HT led to a dramatic reduction in both primary and spontaneous metastatic tumour burden (lungs and bones) with a concomitant elimination of CD133^hi^ CSCs (Fig. 2b–d and Supplementary Fig. 3d).

Consistent with the experimental models, we isolated and cultured patient-derived HTR bone metastases (Supplementary Table 3, HTR-Bone-Met) and determined that these cells produced increased levels of IL6 mRNA in response to fulvestrant (Supplementary Fig. 3e). Importantly, fulvestrant enhanced the self-renewal (growth as secondary MS) of the HTR-Bone-Mets, which was abrogated with the addition of tocilizumab (Fig. 2e,f and Supplementary Fig. 3f).

### IL6R blockade increases ER and reduces Stat3/Notch3/CD133

**Figure 3:**
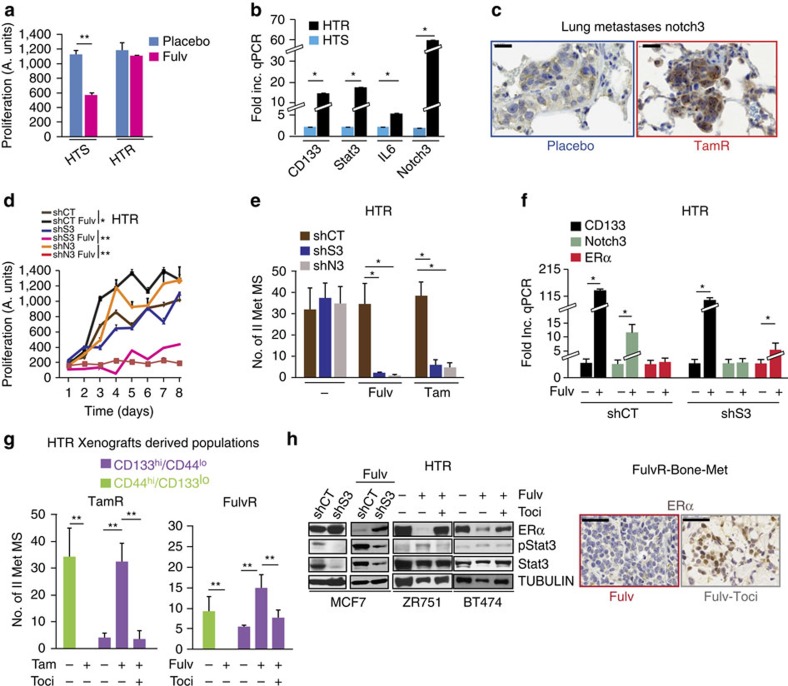
IL6R blockade increases ER and decreases Stat3/Notch3/CD133 expression restoring HT sensitivity. (**a**) Metastatic tumour cells, isolated from Fulv-resistant metastases (MCF7:FulvR, Supplementary Table 2), were cultured in the presence of vehicle (Placebo) or Fulv (10 μM), and growth was determined after 10 days using CalceinAM fluorescent probe: partial (sensitive, HTS) or complete hormone-resistant features (HTR) were generated. Data are reported as mean (fluorescence)±s.e.m. of the last time point of the growth curve (three biological replicates with three technical replicates each). (**b**) Fold increase in the expression of CD133, Stat3, IL6 and Notch3 mRNA using qPCR from HTR and HTS cells treated with Fulv (see **a**, HTS transcript as reference). (**c**) Representative images of Notch3 expression by IHC of lung metastases from placebo versus TamR mice (scale bar, 20 μm). (**d**) Proliferation (CalceinAM fluorescence) in Stat3 or Notch3 stably silenced HTR cells (shS3, shN3) derived from FulvR-Met and propagated *in vitro* (**a**) in the presence/absence of Fulv (10 μM). Data are reported as the mean (fluorescence)±s.e.m. of each time point of the growth curve (three biological replicates with three technical replicates each). (**e**) Number-No- of II-MS (>100 μm) from FulvR-metastatic cells (MetMS) expressing stable shS3 and shN3 cultured in the presence/absence of Fulv (10 μM) and/or tamoxifen (1 μM, 14 days). (**f**) qPCR of CD133, Notch3 and ER mRNA (fold increase Fulv versus untreated cells) in shS3-FulvR-metastatic cells cultured in the presence/absence of Fulv *in vitro* (10 μM, 7 days). (**g**) Number of II-MS (diameter>100 μm) from FACS-isolated (10^3^ cells per well) CD44^hi^ and CD133^hi^ cells from TamR-Met and FulvR-Met (Supplementary Table 2) treated with Tam (1 μM) or Fulv (10 μM) and tocilizumab (50 μg ml^−1^), either alone or in combination (7 days). (**h**) Western blot analysis and IHC images for ER expression from HTR cancer cell lines (ZR751, BT474) and xenografts (MCF7) following treatment with Fulv (10 μM, 14 days) in presence/absence of tocilizumab (50 μg ml^−1^, treatment for 14 days) and after shS3 (scale bar, 50 μm). Data are reported as mean±s.d. of three independent experiments (*n*=3; **b**,**e**,**f**,**g**). *P* values (*P<0.05, **P<0.0001) refer to *t*-test (**a**,**b**,**f**), multiple comparisons corrected *t*-test after GLM ANOVA (**e**,**g**) and GLM for repeated measures (**d**).

To determine whether HT-resistant metastases could re-acquire HT sensitivity *in vitro*, individual FulvR metastases were cultured in the presence of HT (tamoxifen or fulvestrant) for 2 months. Some (2/25) of the metastases remained completely resistant to HT (HTR), while the majority exhibited partial sensitivity to fulvestrant but remained resistant to tamoxifen (Fig. 3a and Supplementary Fig. 4a, HTS).

Molecularly, HTR-Mets expressed higher levels of pStat3 and reduced levels of pHer2 and ER as compared with the HTS ones (Supplementary Fig. 4b). In addition, HTR-Mets had increased levels of the IL6-regulated component of Notch signalling—Notch3—together with a higher mRNA expression of IL6, Stat3 and CD133 (Fig. 3b,c). Finally, CD133^hi^ tumour cells (enriched in areas with stromal infiltrates) also expressed Notch3 protein and, using gene set enrichment analysis, CD133^hi^ cells compared with CD133^lo^ cells had elevated mRNA levels of transcripts downstream of Notch signalling (Supplementary Fig. 4c,d, GSE69280).

To determine the functional role of Stat3 and Notch3 in HT resistance, we reduced their levels by stable short hairpin RNA (shRNAs; shS3/shN3) that restored fulvestrant and tamoxifen sensitivity to the HTR cells *in vitro* (both two-dimensional and three-dimensional growth) without affecting growth in the absence of HT (Fig. 3d,e). Although the knockdown of Stat3 did not decrease CD133 mRNA expression, ShS3-HTR cells (cultured with fulvestrant) had reduced Notch3 mRNA levels, suggesting that Notch3 is downstream of Stat3 and re-expressed ER mRNA (Fig. 3f). These latter observations led us to hypothesize that ER re-expression could be a possible mechanism underlying restoration of endocrine sensitivity with the blockade of IL6 signalling.

To test this hypothesis, CD44^hi^ and CD133^hi^ cells were isolated from HT-resistant metastases (TamR and FulvR, from multiple sites Supplementary Table 2) and grown as secondary MSs (II MetMS) with and without HT (Tam or Fulv) and tocilizumab. CD133^hi^ cell growth was actually enhanced with HT, while the addition of tocilizumab reversed these effects, demonstrating that autocrine IL6 is required for the self-renewal of CD133^hi^ cells (Fig. 3g). Conversely, we demonstrated that MS growth from CD44^hi^ CSCs was completely inhibited by HT, supporting the concept that CD133^hi^ and CD44^hi^ are distinct CSCs. At the molecular level, the abrogation of CD133^hi^ self-renewal with combined HT and tocilizumab led to ER protein re-expression in several models of experimental HT-resistant breast cancer occurring in a Stat3-dependent manner (Fig. 3h-full blots in Supplementary Fig. 8). Taken together, we describe a model of HT-driven metastatic disease via the expansion of CD133^hi^/ER^lo^/IL6^hi^ CSCs; we determined the molecular machinery underlying this process and propose a therapeutic intervention reversing HT resistance (HT/tocilizumab).

### HT induced a dormant CD133^hi^/OXPHOS^lo^/Mito^lo^ phenotype

**Figure 4:**
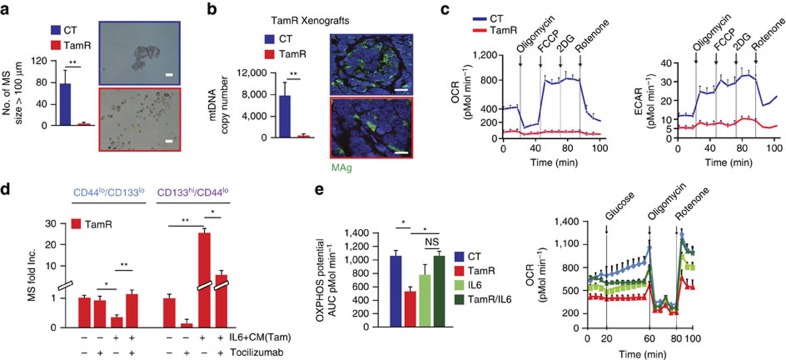
HT induces a dormant CD133^hi^/OXPHOS^lo^/Mito^lo^ phenotype. (**a**) Graph of MS number (diameter>100 μm) generated from FACS-isolated CD44^lo^/CD133^lo^ MCF7 tumour cells treated with tamoxifen (1 μM, TamR cells) for 2 months, representative images of cultured cells in 3D (scale bar, 50 μm). (**b**) Quantification of mtDNA (see Methods) in tumour-derived tissue and cells isolated from TamR xenografts (from Fig. 1g). Representative immunofluorescence of MAg expression in TamR tumours (scale bar, 15 μm). (**c**) OCR and ECAR of cancer cells described in **a** and treated with mitochondrial stressors (oligomycin 1 μM, FCCP 300 nM, 2DG 50 mM, Rotenone 300 nM, see Methods). (**d**) MS growth (fold increase in the number of MS>100 μm; as a reference we used non-treated cells) in TamR-derived CD44^lo^/CD133^lo^ and CD133^hi^/CD44^lo^ cultured in IL6-containing conditioned media derived from tamoxifen-treated cells (CM-Tam) in the presence/absence of tocilizumab (50 μg ml^−1^, 7 days). (**e**) OCR was measured in cells following treatment with Tam (1 μM, 2 weeks), IL6 (10ng ml^−1^, was added 24 h before analysis) and IL6/Tam in the absence or presence of mitochondrial stressors (0.5 μM oligomycin, 100 nM rotenone—doses leading to partial inhibition of OXPHOS potential) and in presence of high glucose (20 mM). Bar graph representing the median AUC±s.d. area under the curve of OCR, pMol min^−1^, see Methods (three biological replicates with three technical replicates each). Data are reported as mean±s.d. of three independent experiments (*n*=3; **a**,**b**,**d**). *P* values (*P<0.05, **P<0.0001) refer to *t*-test (**a**,**b**) and *post hoc t*-test after GLM ANOVA (**d**,**e**).

Given the critical role of CD133^hi^ cells in the development of HTR disease, we sought to determine their provenance. CD133^hi^ cells are enriched in tumours from HT-treated mice, which also have reduced ER expression (Fig. 1c and Supplementary Fig. 4c); we therefore hypothesized that reducing ER activity in differentiated cells (with tamoxifen) could convert CD133^lo^ cells into CD133^hi^ ones. We treated CD133^lo^/CD44^lo^ cells with tamoxifen for 2 months, and the surviving TamR cells (DAPI-negative) were enriched 100-fold for CD133^hi^/CD44^lo^-expressing cells (tamoxifen-resistant cancer cells, TamR) and conversely were reduced in CD44^hi^-expressing cells compared with vehicle-treated (control) cells (Supplementary Fig. 5a–c). However, these CD133^hi^/CD44^lo^/TamR cells did not proliferate nor self-renew (Fig. 4a), lacked features of senescent cells (beta-galactosidase-negative) and were arrested in G2/M of the cell cycle (Supplementary Fig. 5d). In agreement with these functional observations, analysis of tissues from TamR xenografts revealed reduced mitochondrial DNA copy number (mtDNA) and reduced expression of mitochondrial antigen (MAg, a surrogate for mitochondrial mass; Fig. 4b)^19^. Further analysis demonstrated that CD133^hi^/ER^lo^-expressing cells also have reduced MAg expression (Supplementary Fig. 5e). In line with these observations, xenograft-derived fluorescence-activated cell sorting (FACS)-sorted CD133^hi^/CD44^lo^ cells had reduced expression of enzymes regulating lipidogenesis and glycolysis using microarray analysis (PMK-2, LDHA, ACACA, FASN, ACLY, ALDOC and ENO2) as compared with the CD133^lo^/CD44^lo^ cells (Supplementary Fig. 5f, GSE69280). Consistent with the loss of mtDNA/mass, TamR cells had reduced mitochondrial activity (Fig. 4c, as measured by reduced oxidative phosphorylation (OXPHOS) and extracellular media acidification potential). To demonstrate a functional link between the loss of mitochondrial bioenergetics and the generation of CD133^hi^ CSCs, we cultured CD133^lo^/CD44^lo^ cells (from multiple luminal breast cancer models: T47D, BT474 and ZR751) in media containing ethidium bromide, which eliminates mitochondrial DNA (MitoS media, see Methods, Supplementary Fig. 6a). Similarly to what was observed with tamoxifen treatment, the growth of luminal breast cancer cells in MitoS media led to a 20- to 100-fold increase in CD133^hi^ cells (Supplementary Fig. 6b). Likewise, xenograft-derived MCF7 CD133^lo^/CD44^lo^ cells grown in the presence of MitoS media also demonstrated a reduction in CD44^hi^ cells and a 100-fold increase in CD133^hi^/CD44^lo^ cells, which could not self-renew (Supplementary Fig. 6c,d). Notably, MitoS cells not only expressed high levels of CD133 mRNA but also the IL6R and reduced levels of ER mRNA (Supplementary Fig. 6e). Overall, these data suggest that the suppression of OXPHOS activity (MitoS) generates self-renewal-deficient CD133^hi^/ER^lo^ cells.

We then hypothesized that IL6 could drive the exit from HT-induced dormancy of these CD133^hi^/ER^lo^/Mito^lo^ cells by restoring mitochondrial activity in an ER-independent manner (for example, during or following chronic suppression of ER activity). IL6 from the conditioned media of tamoxifen-treated cancer cells (CM-IL6+Tam) mediated the self-renewal capacity of TamR-FACS-isolated CD133^hi^ cells, demonstrating the sufficiency of this cytokine in promoting exit from metabolic dormancy, while the same conditioned media promoted the growth arrest of CD133^lo^ cells (Fig. 4d). We then demonstrated that IL6 administration of tamoxifen-treated cells selectively reversed tamoxifen-mediated inhibition of mitochondrial activity (OCR, oxygen consumption rate) including cells treated with mitochondrial stressors (Fig. 4e, rotenone and oligomycin).

### Exit from metabolic dormancy and HTR via IL6/Notch3

**Figure 5:**
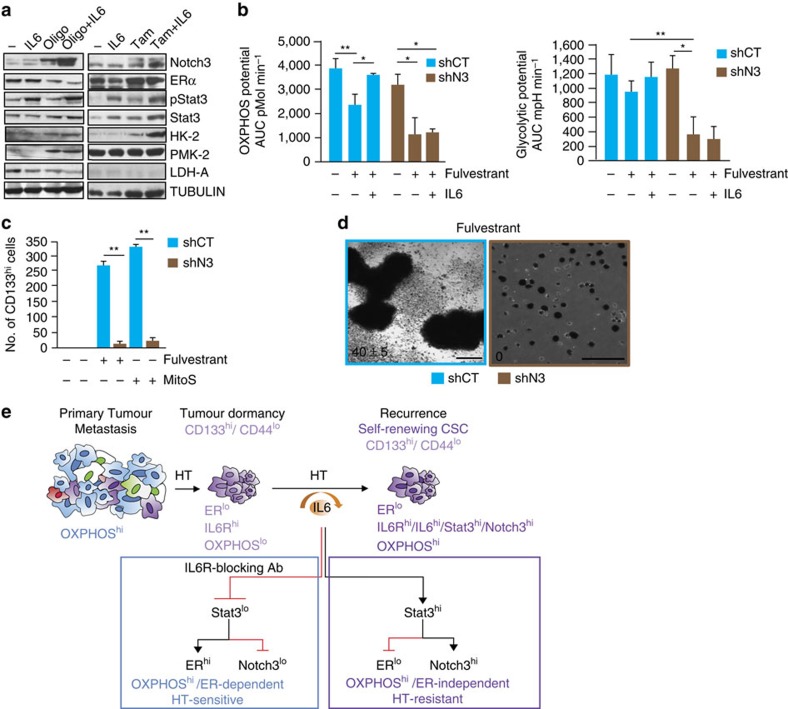
ER-independent exit from metabolic dormancy and HTR disease require IL6/Notch3 upregulation. (**a**) Western blot analysis of Notch3, ERα, pStat3, Stat3, HK-2, PMK-2, LDHA and Tubulin from extracts of MCF7 cells treated with oligomycin (Oligo, 1 μM) or Tam (1 μM) in the presence/absence of exogenous IL6 (10 ng ml^−1^, 24 h, see Fig. 4e). (**b**) OXPHOS and glycolytic potential [AUC of OCR (pMol min^−1^) and ECAR (mpH min^−1^) following mitochondrial stress, see Methods] of HTR shCT and shN3 cells treated with Fulv (10 μM) with/without exogenous IL6 (10 ng ml^−1^, 24 h). Bar graph represents the median AUC±s.d. [AUC of OCR (pMol min); three biological replicates with three technical replicates each]. (**c**) Number of CD133^hi^ cells (by Flow analysis) derived from HTR shCT and shN3 cells treated with Fulv (2 months) or MitoS media (2 months, see Methods). (**d**) Representative image of MS grown from HTR (shCT and shN3) cells in the presence of Fulv (10 μM, 7 days; scale bar, 100 μm). (**e**) Schematic representation of our experimental model describing metastatic progression in luminal breast cancer. Primary tumour cancer stem cells rely on ER expression/signalling for their self-renewal. HT leads to a reduction in ERα levels and activity promoting a dormant cancer stem cell phenotype (CD133^hi^/ER^lo^), characterized by increased IL6R expression and decreased mitochondrial activity. Persistent loss of ERα function promotes the survival and self-renewal of CD133^hi^/Notch3^hi^/ER^lo^ CSC, which occurs in an IL6 (autocrine)-dependent manner and an ER-independent manner. IL6/Stat3 blockade (tocilizumab) restores ERα expression in tumour cells and suppresses the generation of CD133^hi^ cells and disrupts the establishment of an IL6^hi^/ERα^lo^/Notch3^hi^-positive feed-forward loop, preventing the self-renewal of HTR CD133^hi^ CSCs. Data are reported as mean±s.d. of three independent experiments (*n*=3; **b**,**c**). *P* values (*P<0.05, **P<0.0001) refer to *t*-test (**c**) and *t*-test after GLM ANOVA (**b**).

Further analysis of cancer cells treated with mitochondrial stressors and/or tamoxifen demonstrated that IL6 treatment reduced cell death (Supplementary Fig. 7a) and led to an increased expression of Notch3 and enzymes of aerobic glycolysis such as HK-2, PMK-2 and PDH-A (Fig. 5a, full blots Supplementary Fig. 9). In addition, IL6 treatment of these cells promoted the localization of Notch3 to the mitochondria by co-immunofluorescence of Notch3 and Mitotracker and by western blot analysis (Supplementary Fig. 7b,c). This led us to hypothesize that restoration of OCR by IL6 in ER^lo^/OXPHOS^lo^ cells could be dependent on Notch3. To test this hypothesis, we isolated HTR cells (that is, from fulvestrant-treated and resistant tumours) and silenced Notch3 expression (shN3; Supplementary Fig. 7d). We demonstrated that shN3 cells re-acquired sensitivity to HT (Supplementary Fig. 7e), as fulvestrant treatment inhibited their growth and reduced OXPHOS/glycolysis, which was not rescued by IL6 administration (Fig. 5b). In addition, by reducing Notch3 expression, the generation of CD133^hi^ cells was virtually eliminated with concomitant suppression of ER (fulvestrant) and/or mitochondrial activity (MitoS media, see Methods), suggesting that Notch3 expression is crucial for the survival of CD133^hi^ cells following HT and/or mitochondrial stress (MitoS; Fig. 5c). Finally, the cells that survived HT/Notch3 reduction (fulvestrant/shN3) were characterized by self-renewal-deficient non-proliferating single cells in 3D culture (Fig. 5d). Our data suggest that the development of HTR involves the impairment of mitochondrial bioenergetics (low mitochondrial mass-Mito^lo^) and the upregulation of the CSC marker CD133 (CD133^hi^/Mito^lo^). Furthermore, on suppression of ER-dependent mitochondrial respiration (with HT), the IL6/Notch3 pathway promoted the self-renewal of HT-therapy-induced dormant CD133^hi^/ER^lo^/Mito^lo^ cells in an ER-independent manner (Fig. 5e).

## Discussion

Although adjuvant HT is aimed at targeting ER+ metastatic disease, HT is not curative^20^. Thus, an understanding of the molecular underpinnings of metastatic disease and HT resistance is essential to both prevent and treat this fatal disease. Because there are no metastatic models that encompass the pathological progression of ER+ breast cancer, we focus on inhibiting pathways that are critical for primary tumour growth rather than targeting the cell types/pathways that mediate hormone-resistant metastasis. Thus, development of novel therapies would be greatly facilitated by preclinical tumour models that recapitulate the disease process.

Here we established an experimental model of HT-resistant metastatic disease and demonstrated that chronic suppression of ER led to reduced expression of ERα and conversely increased expression of autocrine IL6, selectively in CD133^hi^ cells, driving the self-renewal capacity of these cells in HT-resistant metastases. Importantly, these same phenomena were observed in patient-derived HT-resistant bone metastases. Specifically, HT enhanced MS-forming capacity of these cultured cells with concomitant increases in IL6 expression. In addition, CD133^hi^ expression was increased in patient-derived HT-resistant metastases as compared with primary tumours. Importantly, combination HT and anti-IL6R (tocilizumab) therapy effectively restored HT sensitivity via the re-expression of ERα and abrogation of CD133^hi^ self-renewal in both the preclinical models and in human specimens.

In addition, we identified the stepwise generation of a mitochondrial-defective/therapy-induced dormant population—CD133^hi^/ER^lo^/Mito^lo^—generated through the actions of HT via the suppression of OXPHOS, which reduces ER expression and conversely upregulates CD133^hi^-expressing cells. Chronic HT also induced high levels of paracrine IL6 expression. Mechanistically, we then demonstrated that these metabolically dormant cells (CD133^hi^/ER^lo^/Mito^lo^) exit quiescence via an IL6/Stat3/Notch3-mediated induction of mitochondrial activity.

Importantly, our results are the first to establish a truly representative spontaneous model of ER+ breast cancer, with powerful implications in furthering our understanding the mechanisms underlying the process of HT-mediated dormancy, resistance and metastatic progression to bone and other organs. Finally, our data support the use of targeted therapies against IL6 (anti-IL6R therapy) in patients who have developed HT-resistant disease, which we propose will block the IL6^hi^/ER^lo^ feed-forward loop underlying the *de novo* expansion of CD133^hi^ CSCs and metastatic progression (Fig. 5e).

## Methods

### Establishment of endocrine-resistant metastatic disease model

Primary tumour and metastatic cells were collected from long-term xenografts generated following repeated orthotopic injection in the mammary fat pad of non-obese diabetic/severe combined immunodeficiency mice (NOD/SCID) mice. Cells were isolated and directly stained for FACS analysis or were sorted by green fluorescent protein (GFP) using antibodies against CD44 and CD133 (DAPI-negative). FACS-isolated tumour cells were re-transplanted into recipient mice. All the *in vitro* experiments were generated using tumour-derived cancer cells from xenografts and primary human ductal carcinoma tissues. Preclinical therapeutic trials were generated using xenografts from tumorigenic MCF7 clones administered with tamoxifen citrate implants, fulvestrant (Faslodex) and tocilizumab (ACTEMRA).

### Cell lines and drugs and preclinical trials

MCF7, ZR75, T47D and BT474 breast cancer cell lines were purchased from the American Type Culture Collection (ATCC). All cancer cell lines (ATCC) were mycoplasma free, engineered to express a GFP-positive luciferase expression vector, maintained in MEM/RPMI media supplemented with 5% fetal bovine serum, 2mM glutamine, 100 units ml^−1^ penicillin and 0.1mg ml^−1^ streptomycin (Media Core). Before *in vivo* inoculation, cancer cells were FACS-sorted (for GFP) and injected bilaterally in the mammary fat pads of 5–7-week-old NOD/SCID (obtained from NCI Frederick, MD). For each *in vivo* experiment, cancer cells were mixed with an equal volume of Matrigel (BD Biosciences) in a total volume of 50 μl. Bioluminescence (Xenogen, Ivis System) was used to monitor both tumour growth (weekly) and metastatic burden (at necropsy). Cancer cells from primary and metastatic sites were isolated by enzymatic digestion (Collagenase/Hyaluronidase), sorted (GFP+/DAPI−) and cultured *in vitro*. Metastatic disease occurred in mice with or without the surgical removal of the primary tumours in presence of tamoxifen or fulvestrant. For immunostaining assays, organs were collected and fixed overnight in 4% paraformaldehyde, washed, embedded in paraffin and sectioned (Histo-Serve Core). Haematoxylin and eosine staining was performed using standard methods. For the detection of metastases at secondary sites, we performed GFP and ER double immunofluorescence/immunohistochemistry staining (Supplementary Table 4). All the surgical procedures and animal care followed the institutional guidelines and an approved protocol from our Institutional Animal Care and Use Committees at MSKCC. For all the *in vitro* studies we used tumour cells isolated from primary and metastatic lesions from xenografts using FACS (GFP). The following reagents, 4-hydroxytamoxifen tamoxifen, fulvestrant, rotenone, oligomycin, 2 deoxy-D-glucose (2DG) and IL6, were purchased from Sigma-Aldrich, and trastuzumab was purchased from Genentech. For the preclinical studies, we implanted tamoxifen citrate pellets (5 mg per pellet for mouse, Innovative Research of America) between the scapulas of mice bearing primary MFP tumours. Injectable fulvestrant (Faslodex, AstraZeneca) was given intramuscularly in the tibialis posterior/popliteal muscles (1 mg per injection twice a week) for 2 months. Tocilizumab (Actemra, Roche Pharmaceuticals) was diluted in PBS at a final concentration of 20 mg ml^−1^. A dosage of 100 μg g^−1^ per mouse was administered intraperitoneal every week. Control mice received isotype control (placebo). The beta-galactosidase kit was purchased from Cell Signaling; cells were stained according to the manufacturer's protocol. Cell cycle analysis using PI staining was performed in the Flow Cytometry Core at our institution according to their protocol. Primary tumour and metastatic cells (from multiple sites, Supplementary Table 2) were isolated from xenografts, cultured and engineered *ex vivo* to express shRNAs for Notch3 and Stat3, which were described previously^21^,^22^.

### FACS analysis

Tumours were digested in sterile Epicult media (Stem Cell Technology), minced with sterile razor blades and incubated for 3 h in the presence of Collagenase/Hyaluronidase (1,000 units per sample). Cells were washed with sterile filtered PBS supplemented with 1% bovine serum albumin (PBS-BSA 1%) and filtered through a 40-μM nylon mesh (BD Biosciences). For the detection of CD44 and CD133 antigens, cells were stained in a volume of 100 μl (PBS-BSA 1%) with each antibody CD44-APC (100 ng per 10^6^–10^8^ cells, Clone IM7, eBiosciences) and CD133/1-PE (100 ng per 10^6^–10^8^ cells, clone AC133, Miltenyl Biotech). Cells were labelled on ice for 30 min and analysed (BD FACS Aria III, Flow Core). Samples were analysed for cell population distribution and sorted for GFP/viability (GFP+/DAPI−) and CD133/CD44 expression. For flow plot analyses, samples were run using the FlowJo 7.5 software (Tree Star).

### Gene expression and genotypic analyses

For microarray gene expression profiling and real-time PCR (QPCR), we extracted RNA using Trizol (Invitrogen) from FACS-sorted tumour-derived cells. RNA concentration was determined with a NanoDrop 2000. The MSKCC Genomics Core Facility prepared the samples according to the standard protocols and hybridized them on Affymetrix HG-U133 Plus 2.0 microarrays. We processed the data using GC Robust Multi array Average together with updated probe set definitions (hs133phsentrezg). Differentially expressed genes between groups were identified with fold-change and *t*-test cutoffs (GSE69280 array deposited in NCBI, Supplementary Fig. 4d and Supplementary Data 1). For microarray analysis of published data sets, normalized gene expression data were downloaded from the Gene Expression Omnibus. Each gene was mean-centred and scaled by s.d. All analyses were conducted in R. Normalized gene expression data were downloaded from the NCBI for data set GSE10281 (ref. 23; Fig. 1a). A paired, one-tailed, *t*-test was used to determine whether there was an upregulation of CD133 or ER in patients following Letrozole treatment (Fig. 1a). For QPCR, 1 μg of total RNA was reverse-transcribed to cDNA using Superscript III following the manufacturer's protocol (Invitrogen). QPCR for CD133, ER (ESR1), IL6R, IL6, Notch3, CD44 and Stat3 was performed on 250 ng of cDNA using TaqMan pre-custom probes (Applied Biosystems, ESR1: Hs03024789 125 bp; CD133: Hs01009250 75 bp; IL6R: Hs01075666 69 bp; IL6: Hs00985639 66 bp; Notch3:Hs01128541 81 bp; CD44: Hs01075861 70 bp; Stat3: Hs00374280 70 bp) and ABI Prism 7900HT sequence detection system (Applied Biosystems) in accordance with the manufacturer's instructions. For analysis, ΔC_t_ method was applied and fold-change was calculated (2^−ΔΔCt^). All values were normalized to GAPDH expression (TaqMan, Hs02758991 93 bp). Reverse-transcription PCR (RT–PCR) analysis was performed using the following primers: IL6 Forward 5′- GAGAAAGGACATGTAACAAGAGT -3′, Reverse 5′- GCGCAGAATGAGATGAGTTGT -3′; ER; Forward 5′- TGAAAGTGGGATACGAAAAGAC -3′, Reverse 5′- CAGGATCTCTAGCCAGGCACAT -3′; β2μ Forward 5′- ACCCCCACTGAAAAAGATGA -3′, Reverse 5′- ATCTTCAAACCTCCATGA -3′. PCR primers and reagents for RT–PCR were purchased from Invitrogen. Genomic analysis of MCF7 variants is described in Supplementary Table 2 (from HT-resistant metastasis); it was performed by the Molecular Oncology Core of MSKCC: briefly, DNA was extracted from FACS-isolated cancer cells (GFP+) and sequenced for 341 cancer genes (IMPACT analysis).

### Metabolic assays

Glucose uptake was measured with radio-labelled glucose (Deoxy-D-Glucose, 2-[1,2-^3^H(N)], Perking Elmer). Mitochondrial stress media (MitoS) was prepared by adding 2 mM glucose and 1% of serum to the media-specific recipe for generating cells lacking mitochondrial DNA (ρ0 media: 0.4 μg ml^−1^ ethidium bromide, 50 μg ml^−1^ uridine and 100 μg ml^−1^ pyruvate). Loss of mitochondrial respiration and OXPHOS potential in MitoS cells was assessed using the seahorse technology ( www.seahorsebio.com)^24^,^25^. OCR, extracellular acidification rate and glycolytic potential were determined using the seahorse extracellular flux analyser (XF-96, XF-24 Seahorse Bioscience). To allow comparison between experiments, data are presented as OCR (pMol min^−1^) and extracellular acidification rate (ECAR) (mpH min^−1^). Graphs showing the kinetic of OCR and extracellular acidification rate (ECAR) under basal condition followed by the sequential addition of oligomycin (1 μM), *p*-trifluoromethoxyphenylhydrazone (FCCP, 300 nM), 2DG (50 mM), rotenone (300 nM) and glucose (20 mM) are reported (cell mito and glycolysis stress kit from Seahorse Bioscience). The progress curve is annotated to show the relative contribution of basal, ATP-linked (oligomycin) and maximal (FCCP) oxygen consumption and reserve capacity of the cells (after the addition of 2DG+rotenone). Furthermore, OXPHOS and glycolytic potentials were determined by measuring the area of the progress curve after the addition of glucose and rotenone/oligomycin. For *in vitro* labelling of mitochondria, MitoTracker dye was used (Invitrogen), whereas for *in vivo* studies MAg staining was performed. mtDNA was measured by slightly modifying the protocol described in the manuscript from ref. 24. In particular, 20–100 ng of DNA were used in a real-time QPCR using the Kapa probe fast universal QPCR Kit (Resnova) in which an mtDNA fragment (MTND1 gene) and a nuclear DNA fragment (RNASE P-gene) were co-amplified. The mtDNA content was determined as the ratio of the copy number of mtDNA to the copy number of nuclear DNA. For the above experiments, we used primer sequences and probes established in Dr Giuseppe Gasparre's laboratory^26^.

### Protein analysis and *in vitro* studies

For immunoblotting assays, cells were lysed in buffer (50 mmol l^−1^ Tris at pH 7.5, 150 mmol l^−1^ NaCl, 5 μg ml^−1^ aprotinin, pepstatin, 1% NP-40, 1 mmol l^−1^ EDTA, 0.25% deoxycholate and protease inhibitor cocktail tablet, Sigma). Proteins were separated using SDS–PAGE, transferred to polyvinylidene difluoride membranes and blotted with specific antibodies (Supplementary Table 4). For the luciferase assay, cells were plated in six-well plates at a density of 2 × 10^5^ per well. Cells were transfected with 0.3 μg of promoter-luciferase plasmids. pERα and EREtk were a gift from Dr Andre' Tremblay (University of Montreal); pIL6 was a gift from Dr William L. Farrar (The National Cancer Institute, Frederick). To normalize transfection efficiency, cells were also co-transfected with 0.1 μg of the pRL-CMV (Renilla luciferase, Promega). Forty-eight hours after transfection, luciferase activity was measured using the Dual-Luciferase Assay kit (Promega). Three independent experiments were performed, and the calculated means and s.d.'s are presented. For determination of cell viability, we seeded 2,500 cells per well in 96-well plates and treated them with 4-hydroxytamoxifen (Tam, 1 μM), rotenone, oligomycin and MitoS media in presence/absence of IL6 (10 ng ml^−1^). Viable cells were determined 7 days after the treatment using trypan blue and cell counting at the bright-field microscope or DAPI staining at flow cytometer (Dako Cytomation). IL6 enzyme-linked immunosorbent assay (ELISA) was performed using the conditioned medium collected from 5-day cultures of tumour-derived cells seeded at 200,000 cells per plate. Blood samples were drawn through the orbital vein just before killing the mice. Plasma separated from whole blood by centrifugation at 14,000 r.p.m. at 4 °C. Plasma from tumour-bearing mice and the conditioned medium from *in vitro* cultures were then analysed with the human IL6 ELISA kit (BD Biosciences) according to the manufacturer's instructions (SpectraMax plate platform). Proliferation assay was carried out using CalceinAM technology (Invitrogen): cells were seeded in 96-well plates administered with the pre-fluorescent compound for 20 min and fluorescence was read using a plate reader (SpectraMax plate platform). Densitometry of blots was performed using the ImageJ software (National Institute of Mental Health).

### MS formation potential

MS cultures were generated from eight fresh surgical specimens from HTR bone metastases (Supplementary Table 3, USA-MSKCC cohort) and laboratory-derived experimental luminal breast cancer xenografts (HTR following tamoxifen or fulvestrant administration *in vivo*). Briefly, tissues were placed in sterile Epicult (Voden Medical), minced with sterile razor blades and incubated for 8–12 h in the presence of Collagenase/Hyaluronidase (1,000 units) enzyme mix (Voden Medical). Samples were pelleted at 80*g* for 5 min and digested with Dispase and DNAse (Voden Medical). Pellet (450*g* for 10 min) was resuspended and filtered through a 40-μM nylon mesh (Voden Medical). Cell suspension was plated on 3-cm^2^ low-attachment well plate (Corning), filled with 3 ml Mammary Epithelia Growth Medium, supplemented with *ad hoc* aliquots of B27, 10 ng ml^−1^ EGF, 10 ng ml^−1^ bFGF, 10 μg ml^−1^ insulin and 10^−6 ^M hydrocortisone (Voden Medical). Secondary and tertiary MS potential for pre-sorted tumour-derived cells and cell lines (II-MS or III-MS) was performed as follows: 7-day primary MS started to form after 4–6 days, and then they were disaggregated in 1 × Trypsin-EDTA (Stem Cell Technologies), washed in complete Mammary Epithelia Growth Medium, filtered throughout a 40-μm nylon mesh and seeded to form second-generation MS. Number of MS was assessed by counting the total number of spheres (size>100 μm) from cells seeded in low-attachment plates (from 100 to 1,000). For patient-derived tissue, informed consents were obtained from (1) the ethical committee of Wellness after Breast Cancer study, an ongoing cohort study of breast cancer patients who were treated with an aromatase inhibitor at the University of Pennsylvania (University Penn cohort) and (2) from IRB protocol #97-094 (USA-MSKCC cohort), which includes the analysis of patient-derived specimens.

### Immunostaining analysis

Immunohistochemical analysis was performed on paraffin sections from eight HTR-metastatic tissues and matched primary tumours (Supplementary Table 1, University Penn cohort), eight HTR breast cancer bone metastases (Supplementary Table 3, MSKCC cohort) and experimental xenografts (Figs 1). Sections were de-waxed, rehydrated and retrieved using a Tris-EDTA pH 9.0 solution (20 min at 98 °C) for Notch3, CD133 antibodies or Citrate pH 6.0 solution (40 min at 98 °C) for anti-Her2 and anti-ERα antibodies. Endogenous peroxidase activity was quenched with a Methanol/H_2_O_2_ 1.5% solution (20 min at room temperature (RT)). Primary antibodies were incubated overnight at RT in a humid chamber and processed with a non-biotin peroxidase-amplified system (Novolink, Novocastra Lab) according to the manufacturer's instructions. The immunological reaction was visualized with 3-3′-diaminobezidine tetrahydrochloride (DAB)/H_2_O_2_ solution. Sections were counterstained with Harrys haematoxylin, dehydrated and mounted in Bio-Mount (Bio-Optica). When mouse tissue was used, a short treatment (30 min at RT) with the MOM blocking solution (Vector Laboratories Inc.) was conducted before overnight incubation of primary antibodies at 4 °C. Anti-pancytokeratin immunostaining was performed on Ventana Benchmark Ultra immunostainer with antigen retrieval (UltraCC1 at 95 °C), and primary antibody incubation for 8 min at RT and for 32 min at 37 °C. The reaction was visualized using the UltraView DAB Detection System. The entire neoplastic cell population in mouse and human tissues was evaluated at × 200. For each microscopic field, CD133 staining was scored according to both positive percentage and staining intensity as follows: percentage, 0 if <1%; 1 if ⩾ 1 to <5%; 2 if ⩾ 5% to <10%; 3 if ⩾ 10%. Immunostaining for Her2, ER and PgR expression was quantified using image cytometry with the IMAGE ProPlus 5.1 software (Media Cybernetics Inc.). Her2 expression was evaluated by using the Her2 Pathway Ventana Medical Systems software (Inc.Tucsone USA, FDA-approved for breast tissue). For each section, a score was reported (0, 1+, 2+, 3+) in addition to the total number of positive cells (Supplementary Fig. 2). Their product gave the final score. A labelling index was obtained and expressed as the percentage of labelled area over the total neoplastic nuclear area. For immunofluorescent staining cells (chamber slides) and tissues, slides were incubated with primary antibodies (Supplementary Table 4). Secondary antibodies used were horse anti-rabbit or anti-mouse conjugated with Alexa fluor 568/488 (Molecular Probes). Pictures were taken using a Leica up-right confocal microscope (Molecular Cytology core). For *in vitro* labelling of mitochondria, cells were incubated with mitotracker (Invitrogen) according to the manufacturer's instructions. Number of metastases in distant organs (lymph nodes, lungs, liver, peritoneum, bone and adrenal) was estimated by counting GFP/ER double-positive areas on sequential tissue slides of the same tissue (3) using confocal microscopy.

### Statistical analysis

Statistical analysis was performed using SPSS (SPSS Incorporation). Continuous variables were analysed by unequal variance *t*-test, paired *t*-test (samples, *n*=2), general linear model (GLM) ANOVA or GLM for repeated measures (samples, *n*>2). Mann–Whitney, Wilcoxon and Friedman tests were used to analyse ordinal variables. *P* values were adjusted for multiple comparisons according to Bonferroni correction. Association among quantitative variables was quantified by Pearson correlation coefficient. Categorical variables were analysed by Fisher's exact test or Monte Carlo *χ*^2^-test. All the tests were two-sided. *P*<0.05 was considered significant. The Elda software was used to measure the statistics of limiting dilution experiments (Supplementary Fig. 1f)^27^.

## Additional information

**Accession codes:** GSE69280 array deposited in NCBI

**How to cite this article:** Sansone, P. *et al*. Self-renewal of CD133^hi^ cells by IL6/Notch3 signalling regulates endocrine resistance in metastatic breast cancer. *Nat. Commun.* 7:10442 doi: 10.1038/ncomms10442 (2016).

## Supplementary Material

Supplementary Information Supplementary Figures 1-9 and Supplementary Tables 1-4

Supplementary Data Supplementary Data 1. Affymetrix analysis in tumour derived CD133hi/CD44lo vs CD133lo/CD44lo cells (GSE69280)
