# Supplementary material for: Self-renewal of CD133hi cells by IL6/Notch3 signalling regulates endocrine resistance in metastatic breast cancer
Source: Nat Commun. 2016 Feb 9;7:10442. doi: 10.1038/ncomms10442 (PMC4748123; doi:10.1038/ncomms10442)
Supplement: Supplementary Information — Supplementary Figures 1-9 and Supplementary Tables 1-4 [file ncomms10442-s1.pdf]

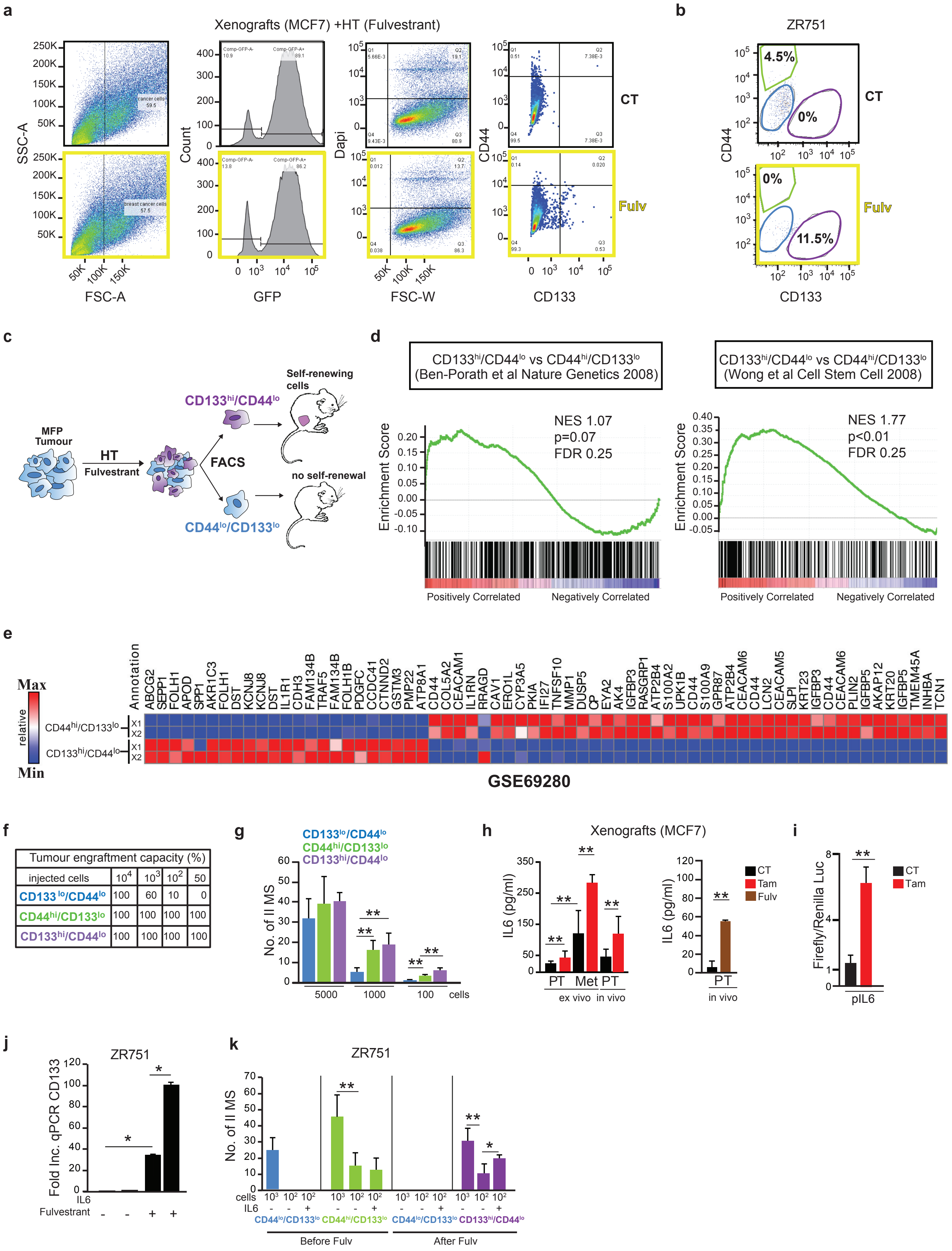
a
Xenografts (MCF7) +HT (Fulvestrant)
SSC-A
FSC-A
Count
GFP
Dapi
FSC-W
CD44
CD133
CT
Fulv
b
ZR751
4.5%
0%
0%
11.5%
CT
Fulv
CD44
CD133
c
MFP Tumour
HT Fulvestrant
FACS
CD133hi/CD44lo
CD44lo/CD133lo
Self-renewing cells
no self-renewal
d
CD133hi/CD44lo vs CD44hi/CD133lo (Ben-Porath et al Nature Genetics 2008)
NES 1.07
p=0.07
FDR 0.25
CD133hi/CD44lo vs CD44hi/CD133lo (Wong et al Cell Stem Cell 2008)
NES 1.77
p<0.01
FDR 0.25
Enrichment Score
Positively Correlated
Negatively Correlated
e
Max
relative
Min
CD44hi/CD133lo
CD133hi/CD44lo
Annotation
ABCG2 SEPP1 FOLH1 APOD SPP1 AKR1C3 FOLH1 DST KCNJ8 KCNJ8 DST IL1R1 CDH3 FAM134B TRAF5 FAM134B FOLH1B PDGFC CCDC41 CTNND2 GSTM3 PMP22 ATP8A1 CD44 COL5A2 CEACAM1 IL1RN RRAGD CAV1 ERO1L CYP3A5 PKIA IFI27 TNFSF10 MMP1 DUSP5 CP EYA2 AK4 IGFBP3 RASGRP1 ATP2B4 S100A2 UPK1B CD44 S100A9 GPR87 ATP2B4 CEACAM6 CD44 LCN2 CEACAM5 SLPI KRT23 IGFBP3 CD44 CEACAM6 PLIN2 IGFBP5 AKAP12 KRT20 TMEM45A INHBA TCN1
GSE69280
f
Tumour engraftment capacity (%)
injected cells 10^4 10^3 10^2 50
CD133lo/CD44lo 100 60 10 0
CD44hi/CD133lo 100 100 100 100
CD133hi/CD44lo 100 100 100 100
g
CD133lo/CD44lo
CD44hi/CD133lo
CD133hi/CD44lo
No. of II MS
5000 1000 100 cells
h
Xenografts (MCF7)
IL6 (pg/ml)
PT Met PT
ex vivo in vivo
CT Tam Fulv
i
Firefly/Renilla Luc
CT Tam
pIL6
j
ZR751
Fold Inc. qPCR CD133
IL6
Fulvestrant
k
ZR751
No. of II MS
cells
IL6
CD44lo/CD133lo CD44hi/CD133lo CD44lo/CD133lo CD133hi/CD44lo
Before Fulv
After Fulv

Fig. S1. HT-resistant tumours express CD133^{hi}/ IL6^{hi} self-renewing CSCs (a) Flow analysis of GFP positive cancer cells isolated from control (CT) and fulvestrant treated (Fulv) MFP tumours (MCF7 xenografts). Cancer cells were harvested from mice bearing MFP tumours after 6 months following (4 months engraftment and 2 months of Fulvestrant/placebo treatment). The rationale for the flow analysis is outlined, including unstained and primary antibody controls (PE, CD133; APC, CD44); **(b)** CD133^{hi}/CD44^{lo} cells were quantified (Flow analysis) from ZR751 tumour-derived cells treated ex-vivo with fulvestrant for 2 months; **(c)** Schematic demonstrating the effect of hormonal therapy on CD133^{hi} CSCs generation *in vivo* and their self-renewal potential; **(d)** Gene set enrichment analysis (GSEA), using publically available stem-cell "signatures" (Ben-Porath et al 2008: ES 0.22,NES 1.07, p=0.07, FDR 0.25; Wong et al 2008: ES 0.39,NES 1.77,p<0.01, FDR 0.25, see methods) was applied to mRNA expression data from FACS sorted CD133^{hi}/CD44^{lo} versus CD44^{hi}/CD133^{lo} tumour-derived cells from 2 different xenografts (p<0.001, t-test cut off); **(e)** Heat map of differentially expressed genes (relative expression) from the expression data described in Microarray experiment (Supplementary Data 1); **(f)** FACS sorted cell populations were isolated from HT-tumours, serially diluted (10^4, 10^3, 10^2, 50 cells) and injected into the MFP and tumour formation was determined (Tumour engraftment capacity, %, n=5 per group). Elda Software was used to measure p value (p < 0.000); **(g)** FACS sorted cells (CD133^{lo}/CD44^{lo}, CD44^{hi}/CD133^{lo}, CD133^{hi}/CD44^{lo} from panel f) were serially diluted (5000, 1000, 100 cells) and the number of secondary mammospheres was determined (No of II MS) for each population; **(h)** IL6 levels from primary tumour (PT) and lymph-node metastases (Met) derived cells treated ex-vivo with tamoxifen (Tam, 1μM); from PT derived cells from mice treated with tamoxifen or fulvestrant (see methods); **(i)** MCF7 cells were transfected with an IL6 promoter-luciferase construct, treated with/without tamoxifen (Tam, 1μM, 24h) and luciferase levels were measured; **(j)** Fold increase of CD133 mRNA expression (y) qPCR in fulvestrant resistant ZR751 cells grown in the presence/absence of exogenous IL6 (10ng/ml for two weeks/weekly administration); **(k)** FACS sorted cells (CD44^{lo}/CD133^{lo}, CD44^{hi}/CD133^{lo}, CD133^{hi}/CD44^{lo}) were isolated from ZR751 tumors (before fulvestrant and after fulvestrant treatment see panel b), serially diluted (10^3, 10^2, 500, 50 cells) and the number of secondary mammospheres was determined (No of II MS) for each population in the absence/presence of IL6 for 2 weeks; Data are shown as the mean ±s.d. of three representative experiments (n=3) (g-k). P values (*<0.05, **<0.0001) refer to t-test (i) and post hoc t-test after GLM anova (g, h, j, k).

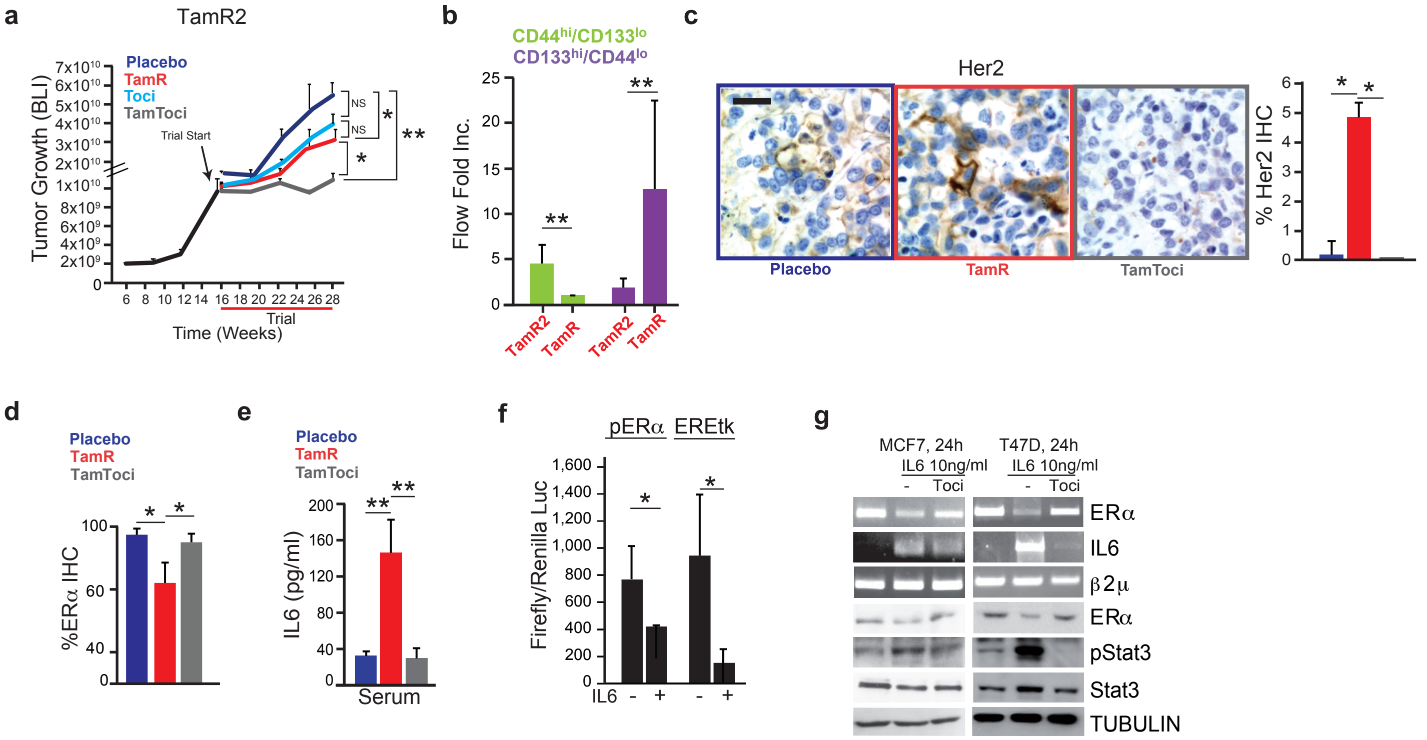

Fig. S2. Tocilizumab reverses tamoxifen resistance via the re-expression of ERα and prevents the generation of CD133^{hi} CSCs **(a)** Representative tumour growth kinetics (by BLI, 31 weeks) of tamoxifen resistant tumours (TamR2). MCF7 (GFP/Luciferase+) xenografts were established after 16 weeks and mice were treated for the following 15 weeks with tamoxifen-citrate pellets (Tam, 5mg/pellet) with/without tocilizumab (Toci, see methods) and in combination -TamToci-. The mean BLI value ± s.e.m is reported for each time point (n=5/group); **(b)** Tumours (from TamR and TamR2 trials) were analyzed by flow cytometry for the percentage of CD133 and CD44 cells; **(c)** Percentage of positive cancer cells with a high Her2 score (3+) from mammary tumours (see methods for quantification) described in Fig. 1g (TamR xenografts). Representative IHC images for Her2 expression is also reported; **(d)** Percentage of ERα positive cancer cells from mammary tumours described in Fig. 1g [tumour bearing mice were treated with vehicle (Placebo), Tamoxifen (TamR) and Tamoxifen/Tocilizumab (TamToci)]; **(e)** Circulating IL6 levels were measured by ELISA from the serum of tumour bearing mice described in Fig.1g (Placebo, TamR, Toci, TamToci); mice (n=5/group) **(f)** T47D cells were transfected with either an ERα promoter (pERα) or an ERα response elements (EREtk) regulated luciferase construct, treated with/without IL6 (10ng/ml, 24h) and luciferase levels were measured; **(g)** mRNA and protein extracts were isolated from MCF7 and T47D cells treated with IL6 (10ng/ml, 24h) in the absence/presence of tocilizumab (Toci, 50μg/ml, 24h). Representative (one of 3) reverse-transcription PCR (RT-PCR) for ERα, IL6, β2μ (beta2 microglobulin) mRNA levels and western blot analysis for ERα, pStat3, Stat3 and tubulin expression are shown. Data are reported as mean ± s.d. of three independent experiments (n=3) (b-f). P values (*<0.05, **<0.0001) refer to t-test (b-f) and post-hoc t-test corrected for multiple comparisons after GLM for repeated measures (a).

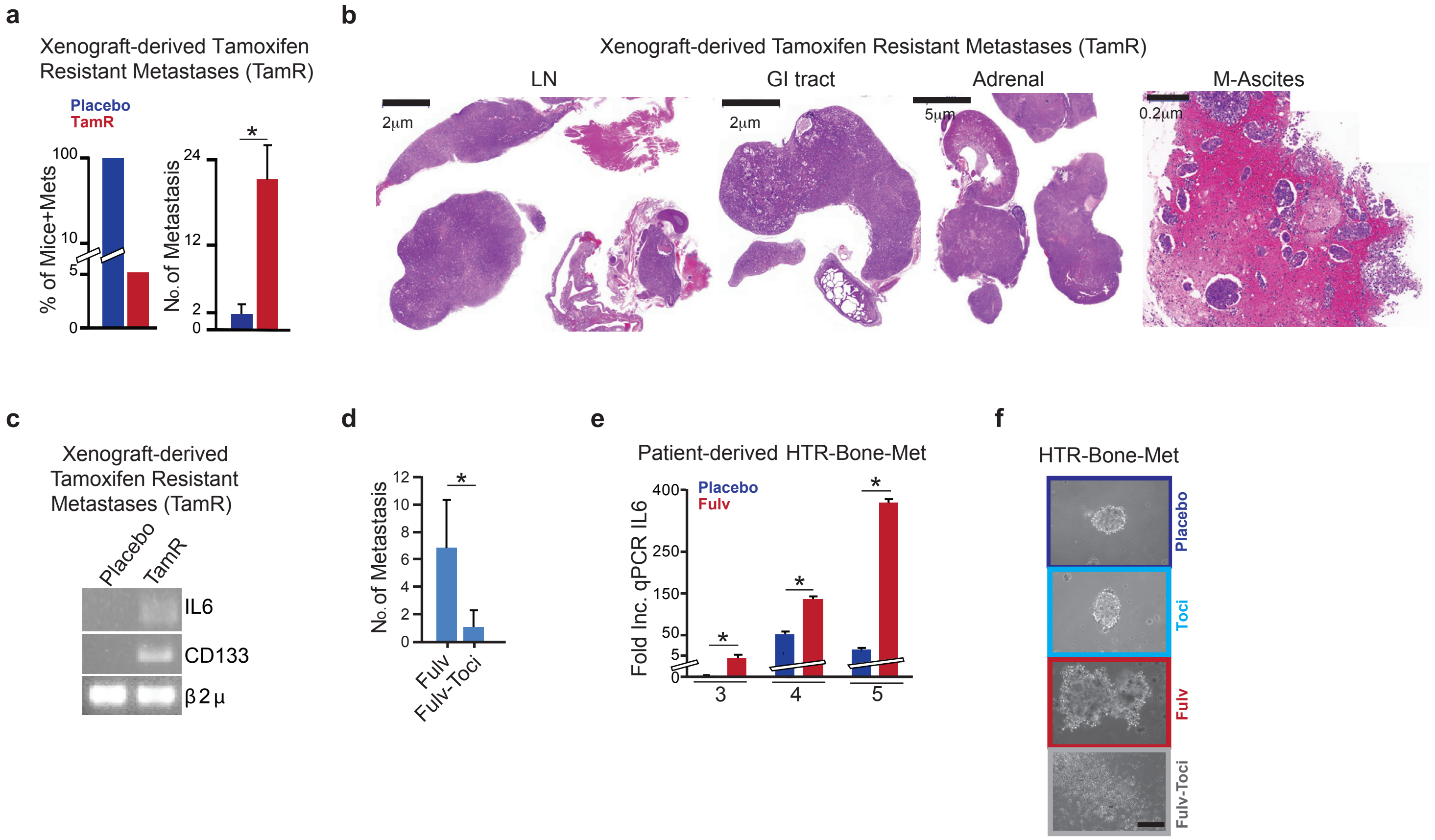

Fig. S3. IL6R-blockade (tocilizumab) re-sensitizes HT resistant metastatic disease to endocrine therapy
(a) Percentage of mice developing metastatic disease after MCF7-primary tumour removal: mice were treated with tamoxifen-citrate pellet after primary tumor removal for 5 months); then the number of mice with metastasis and number of metastases (mean ± s.d.) was determined at necropsy; **(b)** Representative H&E images of metastases from mice bearing tamoxifen resistant metastatic disease (LN, lymph nodes metastasis; GI, metastases in the great curvature of the stomach; Adrenal, adrenal metastases; M-Ascites, malignant ascites); **(c)** mRNA was isolated from FACS sorted cancer cells isolated from tamoxifen resistant (TamR) versus control (Placebo) treated metastases. IL6 and CD133 mRNA levels were determined by RT-PCR and beta2 microglobulin (β2μ) mRNA is reported as quantitative control; **(d)** Number of metastases (by H&E, mean ± s.d. of total number of metastases detected in the experiment of Fig. 2b) was determined from MCF7 xenografts treated with fulvestrant (Fulv) or fulvestrant-tocilizumab (Fulv-Toci). [Fulv vs Fulv-Toci (n=5 mice/group)]. **(e)** qPCR analysis of IL6 mRNA levels of patient derived HTR-B-Mets cultured as mammosphere in the presence/absence of vehicle (Placebo) or fulvestrant (Fulv, 10μM, 7 days) (samples 3, 4, 5 see Supplementary Table 3, mRNA level is presented as fold increase using sample 3 as referenced); **(f)** Representative image of MS growth (sample 8, Scale bar 100μM) of HTR-B-Mets treated with vehicle (Placebo), fulvestrant (Fulv, 10μM, 7 days), tocilizumab (Toci, 50μg/ml, 7 days) and fulvestrant-tocilizumab (Fulv-Toci, 7 days). Data are reported as mean ± s.d. of three independent experiments (n=3) (e). P values (*<0.05, **<0.0001) refer to t-test.

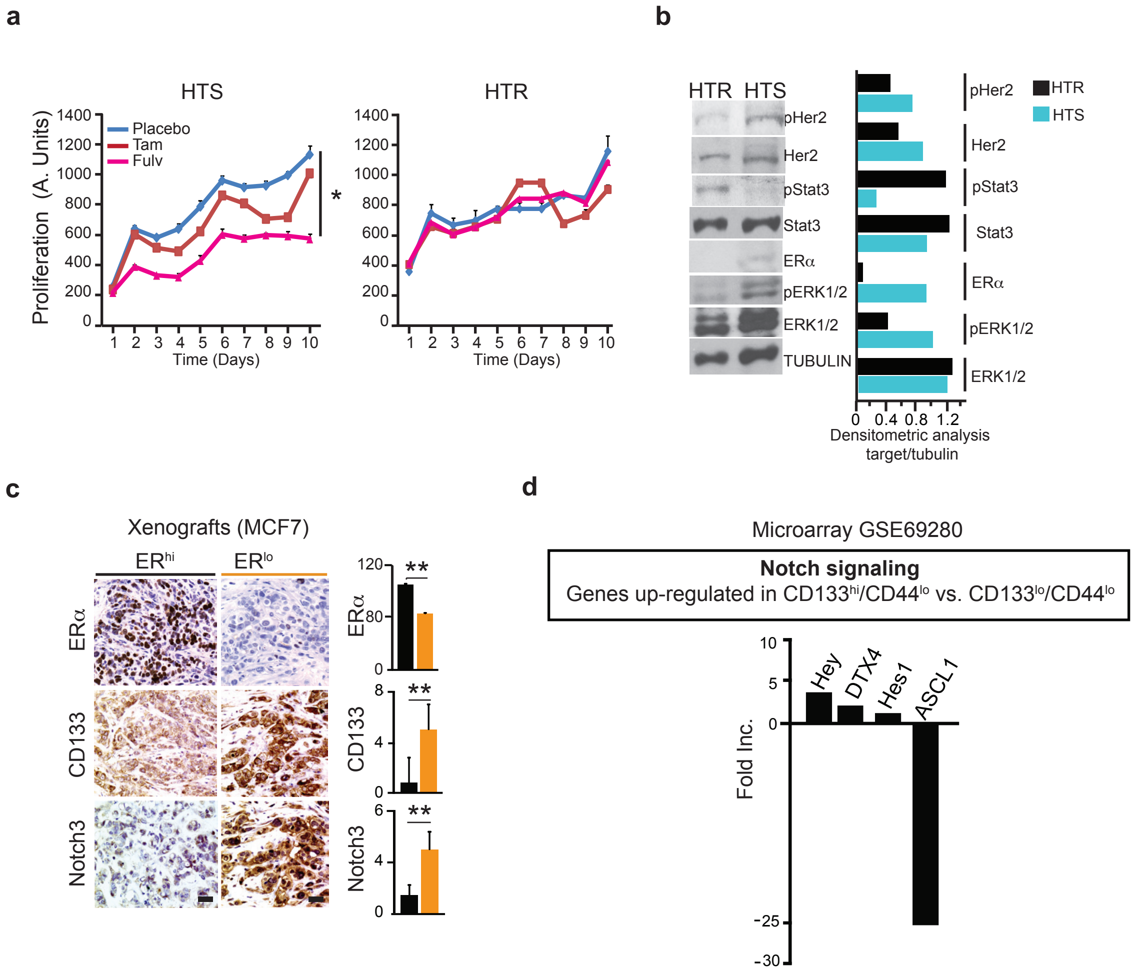

Fig. S4. HTR metastases express a Stat3^{hi}/CD133^{hi}/Notch3^{hi}/ER^{lo} phenotype (a) Metastatic tumor cells, isolated from fulvestrant resistant (FulvR) metastasis (Fig. 3a) were cultured in the presence of vehicle (Placebo), tamoxifen (Tam, 1μM) or fulvestrant (Fulv, 10μM) and growth was determined over time (days, CalceinAM assay). This led to the establishment of metastases with either partial sensitivity (HTS) or complete hormone resistant features (HTR). Two representative examples are shown. Data are reported as mean (fluorescence) ± s.e.m of each time point of growth curve (3 biological replicates with 3 technical replicates each); **(b)** Protein extracts from HTR/HTS cells treated with fulvestrant for 2 weeks (see panel a) were analyzed by western blot for the expression of pHer2, Her2, pStat3, Stat3, ERα, pERK1/2, ERK1/2 and tubulin. Densitometric analysis for expression levels was determined; **(c)** Representative IHC images and quantification of ERα, CD133 and Notch3 expression in tumor tissues from MCF7 xenografts (mass-black) and in areas of desmoplasia (desmo-yellow, scale bar 25μm). Data are reported as mean ± s.d. of n=18 xenograft-derived tissues; **(d)** Fold increase mRNA levels (CD133^{hi}/CD44^{lo} versus CD44^{lo}/CD133^{lo}) of genes related to tumour cell dissemination and Notch signaling (GSE69280, see methods). P values (*<0.05, **<0.0001) refer to post hoc t-test after GLM anova (a) and t-test (c).

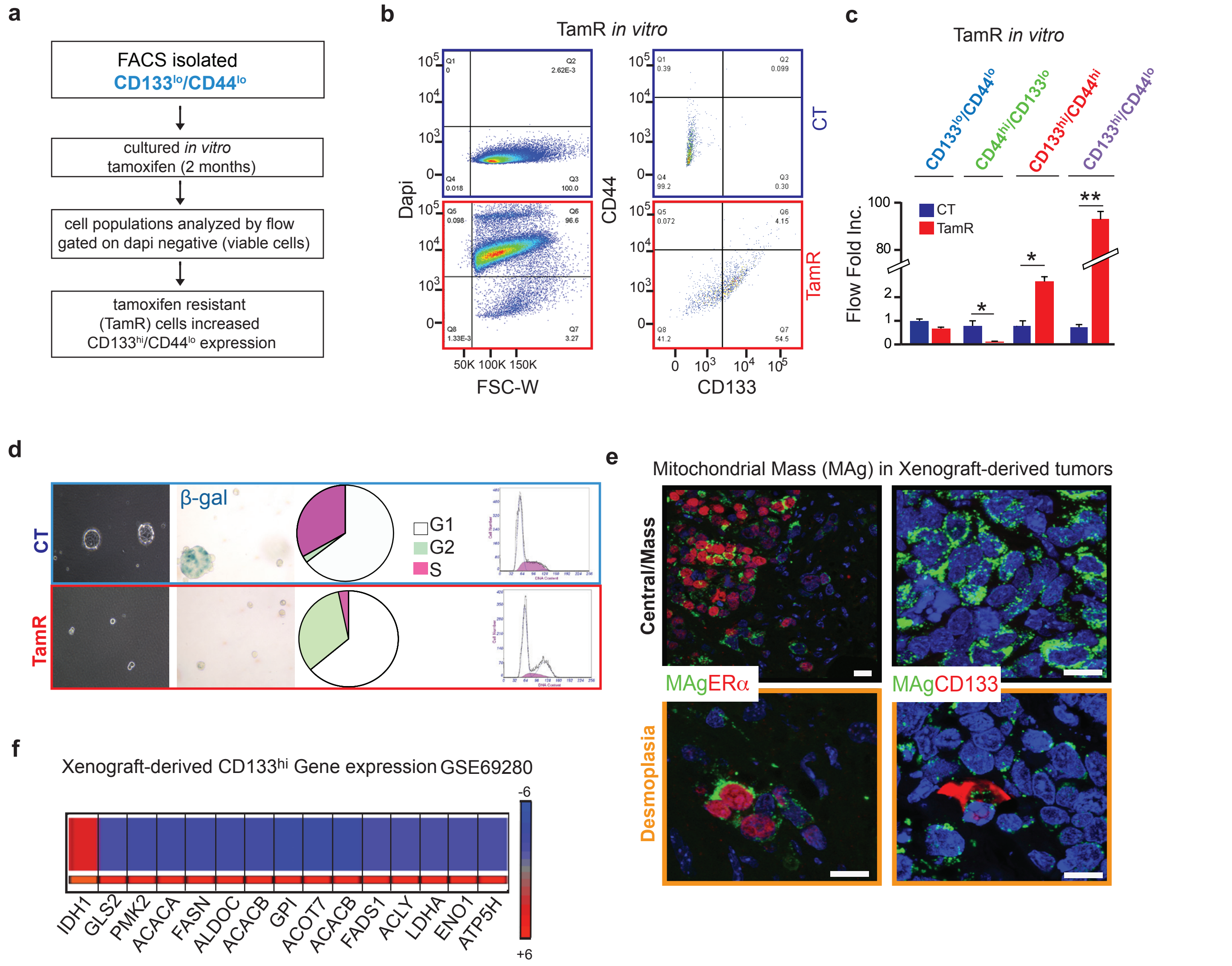

Fig. S5. Metabolically dormant CD133^{hi}/CD44^{lo} cells require increased IL6/Notch3 expression to exit from therapy-induced dormancy (a) Schematic of the experimental design (a), CD133^{lo}/CD44^{lo} cells were FACS isolated from MCF7 xenografts, treated with tamoxifen (1µM, 2 months); tamoxifen resistant cancer cells (TamR, 3% of viable population (**b, c**) were analyzed by flow for CD44 and CD133 expression; **(d)** Cells from panel b TamR and control (CT) were grown as mammospheres: β-galactosidase staining (β-gal) and cell cycle analyses were performed; **(e)** Representative confocal image of mitochondrial antigen (MAg), ERα and CD133 expression from different areas of xenografts tumors [tumour center (mass)-top panels and desmoplasia (desmo)-bottom panels]. Scale bar 15µm; **(f)** Heat map demonstrating the fold change in the expression of lipidogenic and glycolytic genes (mRNA) from xenograft-derived FACS sorted CD133^{hi}/CD44^{lo} versus CD133^{lo}/CD44^{lo} from 2 different xenografts ($p<0.001$, GSE69280, t-test cut off). Data are reported as mean ± s.d. of three independent experiments (n=3) (c). P values (*<0.05, **<0.0001) refer to t-test (c).

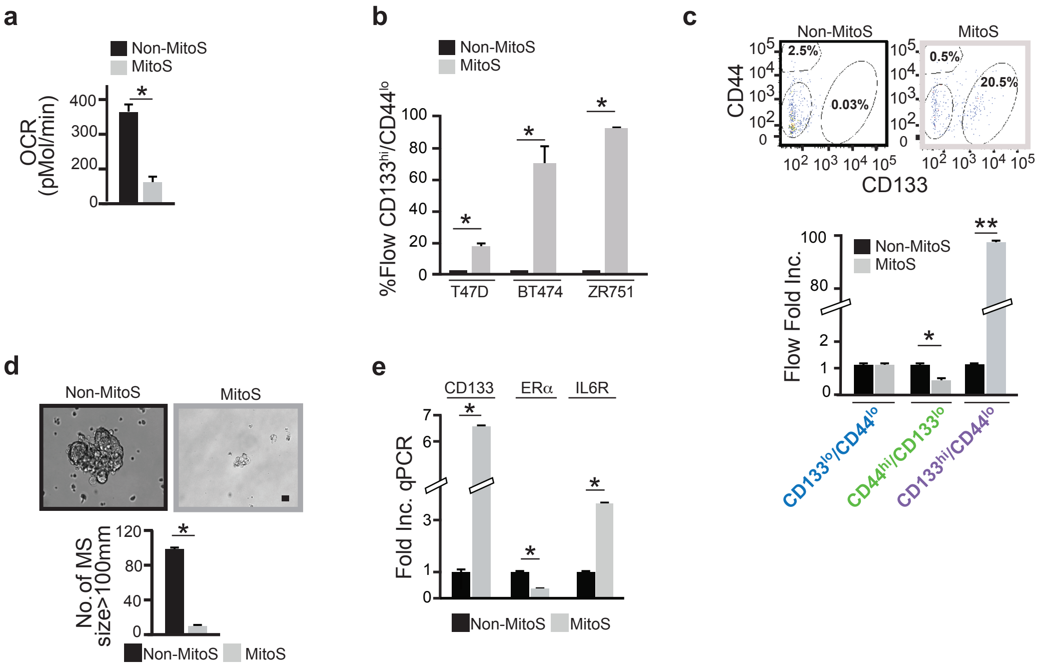

Fig. S6. OXPHOS abrogation promotes the generation of CD133^{hi}/CD44^{lo} cells (a) Representative OCR potential of MCF7 cancer cells undergoing mtDNA depletion with MitoS media (2 months, see methods); **(b)** Percentage of CD133^{hi}/CD44^{lo} cells by FACS obtained from ER+ breast cancer cell lines (T47D, BT474, ZR751) treated with MitoS media or vehicle (Non-MitoS) for 2 months; **(c)** Fold increase of CD133 and CD44 expression by flow cytometry (Flow Fold Inc.) generated from FACS-isolated CD44^{lo}/CD133^{lo} MCF7 tumour cells treated with vehicle (Non-MitoS) or MitoS media for 2 months; **(d)** Number of MS (diameter>100μm) generated from MCF7 tumour cells treated with vehicle (Non-MitoS) or MitoS media for 2 months. Scale bar 25μm; **(e)** qPCR analysis of CD133, ER and IL6R normalized to GADPH levels from MitoS and control (Non-MitoS) cells as described in d. Data are reported as mean ± s.d. of three independent experiments (n=3) (a-e). P values (*<0.05, **<0.0001) refer to t-test (a-e).

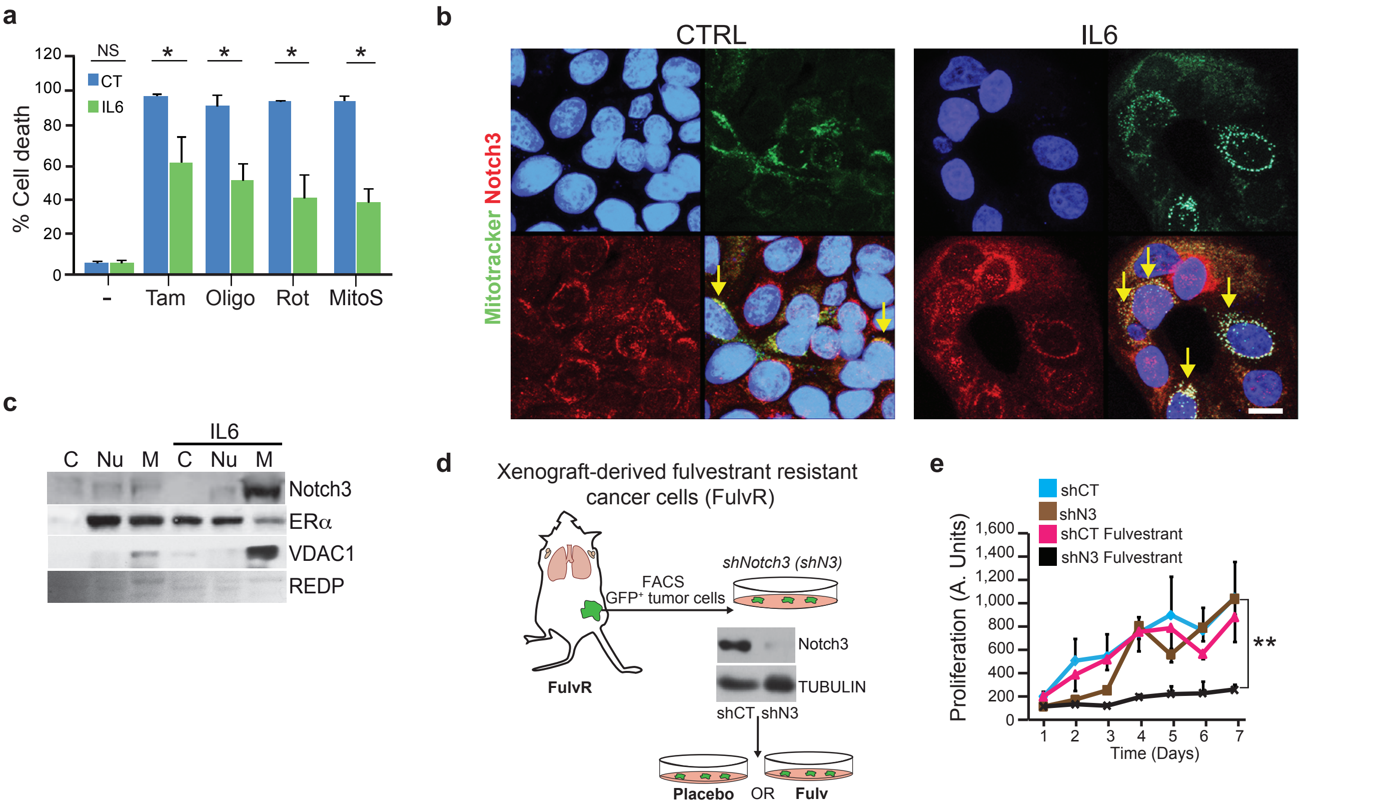

Fig. S7. An IL6 dependent shift from ERα to Notch3 dependent mitochondrial respiration controls hormonal therapy resistance (a) Tumour derived cancer cells were treated with Tamoxifen (Tam,1μM), Oligomycin (Oligo, 1μM), Rotenone (Rot, 100nM) and MitoS media for 14 days and in presence of IL6 (10ng/ml added every 48h), % cell death was determined (Trypan blue). Data are reported as mean ± s.d. of three independent experiments (n=3); **(b)** Representative confocal image of Notch3 (red) immunofluorescence (IF) and MitoTracker (green) from MCF7 cells treated with and without IL6 (10ng/ml for 24h). Translocation of Notch3 in the mitochondria is observed with IL6 (yellow arrows). Scale bar 15μM; **(c)** Extracts from subcellular compartments (C, cytoplasm; Nu, nucleus; M, mitochondria) of MCF7 cells treated with/without IL6 (10ng/ml for 24h) were analyzed by western blot for expression of Notch3, ERα, VDAC1 (a mitochondrial protein) and ponceau red (REDP) as a loading control; **(d,e)** Schematic of the generation of HT-resistant primary tumour cells (HTR, fulvestrant), which were infected in vitro to express an shRNA for Notch3 or control (shN3/shCT cells). Western blot analysis of Notch3 from these cells; shCT and shN3) were cultured in the presence/absence of fulvestrant (10μM, 7 days) and cell proliferation was determined (CalceinAM assay). Data are reported as mean (fluorescence) ± s.e.m of each time point of growth curve (3 biological replicates with 3 technical replicates each). P values **<0.0001 refer to t-test (a) and post-hoct-test corrected for multiple comparisons after GLM anova (e).

Supplementary Figure 8. Uncropped blots for Figure 3h Western Blot Analysis

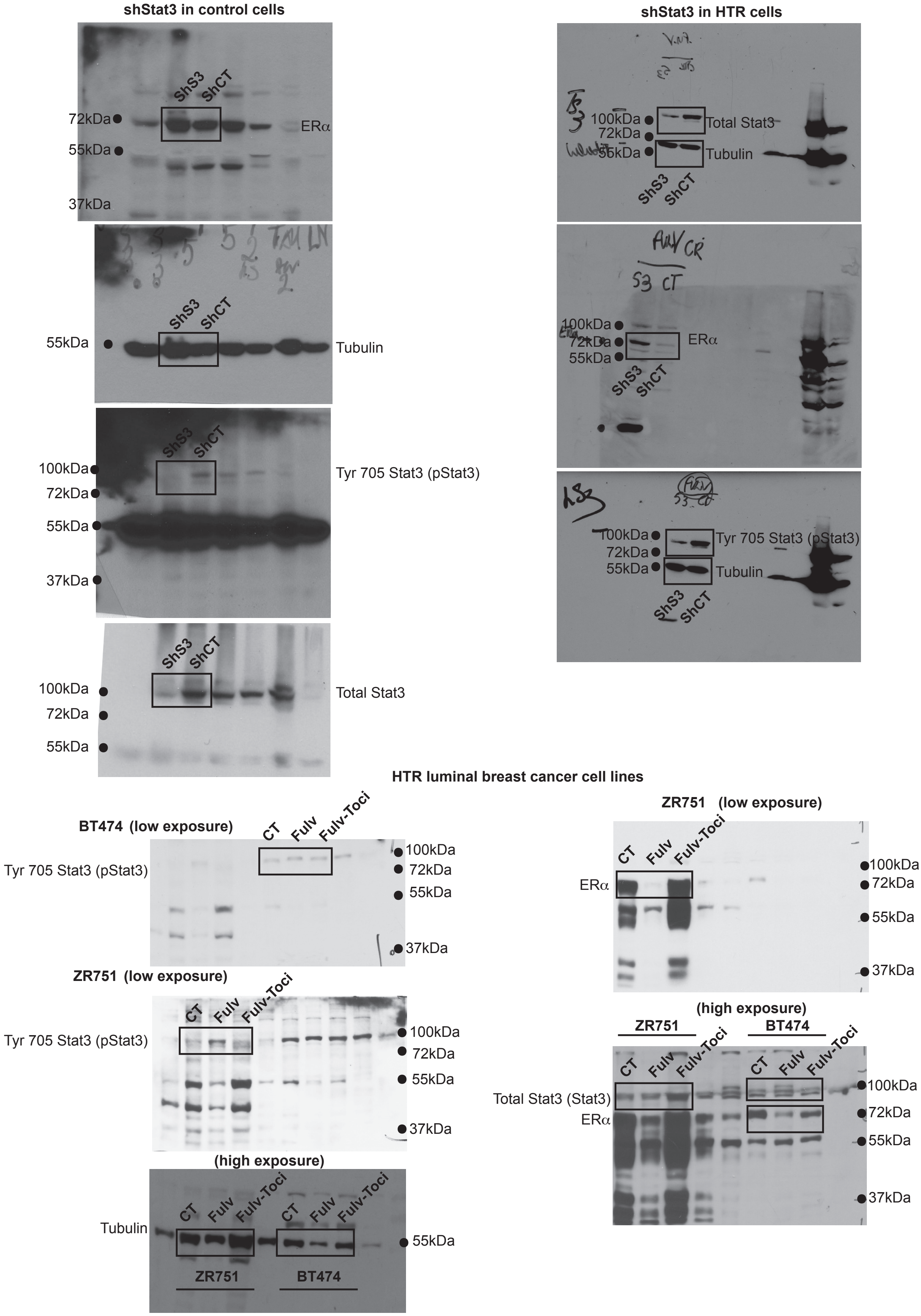

Supplementary Figure 9. Uncropped blots for Figure 5a Western Blot analysis

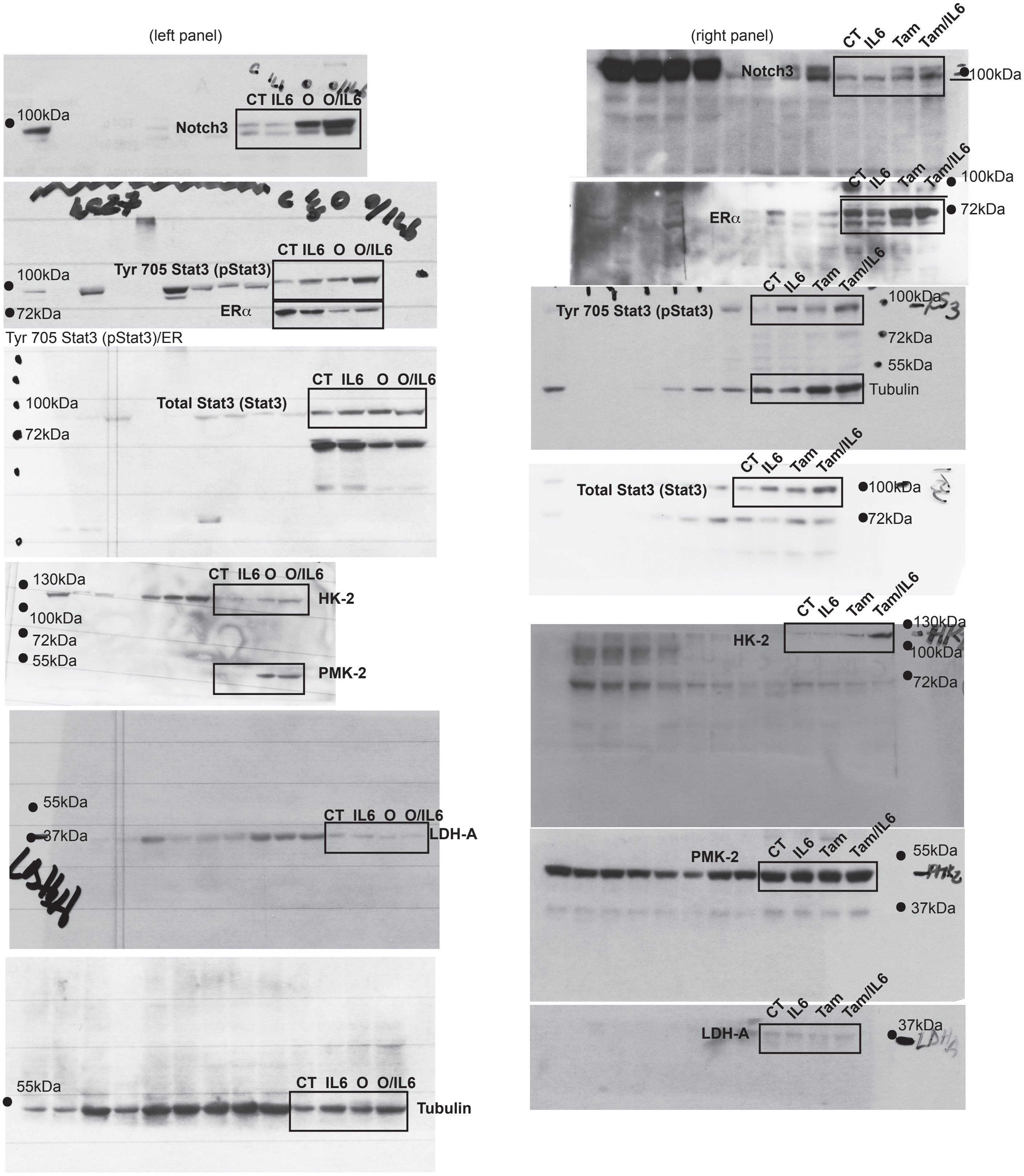

Supplementary Table 1. CD133 IHC score obtained from hormone resistant breast cancer metastasis tissues and matched primary tumours (US-Penn cohort)

Sample ID	Histology	Site of Metastasis	CD133 IHC Score	Endocrine Therapy received
HTR-Met1	Ductal	PT- Right Breast (6/2001)<br>Recurrence to Right Breast (1/2010)	2<br>2	Tamoxifen and Lupron 6/2001 - 4/2004, Aromasin/Lupron 4/2004 - 6/2006
HTR-Met2	Ductal	PT- Left Breast (6/2008)<br>Recurrence to Right Breast (9/2009)	0<br>2	Arimidex 2/2009 - 9/2009
HTR-Met3	Lobular	PT- Left Breast (3/18/2004)<br>Recurrence to Left Axilla (8/2010)	0<br>1	Arimidex 11/2004 - 3/2010
HTR-Met4	Lobular	PT- Right Breast (6/2004)<br>Recurrence to Small Bowel (11/2012)	0<br>1	Femara and Arimidex 7/2004 - 4/2012
HTR-Met5	Ductal	PT- Left Breast (7/2008)<br>Recurrence to Left Breast (11/2012)	1<br>3	Arimidex 10/2008 - 10/2012
HTR-Met6	Lobular	PT- Left Breast (11/1998)<br>Recurrence to Left Breast/Axilla (4/2012)	1<br>1	Tamoxifen 6/1999-8/1999<br>Arimidex 7/03 - 8/08
HTR-Met7	Ductal	PT- Left Breast (11/2008)<br>Recurrence to Chest Wall (10/2010)	1<br>3	Arimidex 3/2009 - 8/2009, Aromasin 8/2009 - 3/2010
HTR-Met8	Ductal	PT- Left Breast (6/2008)<br>Recurrence to Left Ovary (8/2011)	0<br>3	Tamoxifen 2/2009 - 4/2010

Supplementary Table 1. CD133 IHC score obtained in metastatic and primary tissue specimen from hormonal therapy resistant luminal breast cancer patients (HTR-Met, US-Penn Cohort, Fig. 1b).

Supplementary Table 2. Characterization of hormone resistant lesions (HT-R) in the MCF7 experimental model

A. Anatomical Origin of HT-R Metastasis

METASTASES ID	Type of treatment	Site
TAMR1	tamoxifen citrate	Bone Marrow
TAMR2	tamoxifen citrate	axillary LN
TAMR3	tamoxifen citrate	popliteal LN
TAMR4	tamoxifen citrate	lumbar LN
TAMR5	tamoxifen citrate	renal LN
TAMR6	tamoxifen citrate	malignant ascites
TAMR7	tamoxifen citrate	iliac LN
TAMR8	tamoxifen citrate	lung
FULVR1	Faslodex/fulvestrant	Bone Marrow
FULVR2	faslodex/fulvestrant	renal LN
FULVR3	faslodex/fulvestrant	axillary LN
FULVR4	faslodex/fulvestrant	lung
FULVR5	faslodex/fulvestrant	lumbar LN

B. HT-R Metastasis display the same DNA copy number as compared to controls (Met, metastasis; PT, primary tumour TAMR, tamoxifen resistant metastasis; FulvR, fulvestrant resistant metastasis)

PT

Met

TAMR1

FULVR1

FULVR2

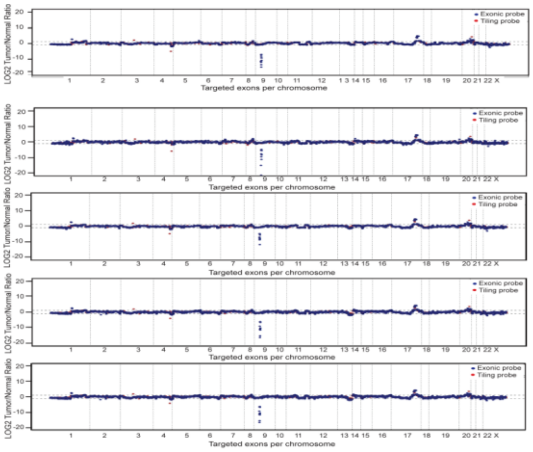

C. HT-R metastasis and controls express the same genomic alterations

Gene	Exon	TranscriptID	cDNA change
BARD1	exon4	NM_000465	c.1075_1095delTTGCCTGAATGTTCTTCACCA
CREBBP	exon11	NM_004380	c.2140C>T
EPHA5	exon18	NM_004439	c.3023T>C
ERBB2	exon17	NM_004448	c.1960A>G
ERBB4	exon28	NM_005235	c.3725A>G
FLT4	exon17	NM_182925	c.2441C>A
GATA3	exon5	NM_002051	c.1000dupG
HIST1H1C	exon1	NM_005319	c.70G>A
MAP2K2	exon3	NM_030662	c.406G>A
MAP3K13	exon6	NM_004721	c.1138G>A
NBN	exon2	NM_002485	c.127C>T
NBN	exon9	NM_002485	c.1090G>A
PIK3CA	exon10	NM_006218	c.1633G>A

Supplementary Table 2. Anatomical origin (A) and genotypic features (B, C) of hormone resistant (HT) metastatic lesions in the MCF7 xenograft model (Lymph-nodes, LN).

Supplementary Table 3. Therapy and clinical features of hormone resistant bone metastases isolated from breast cancer patients (US-MSKCC cohort)

Sample ID	Metastatic Site	Nature of Primary Tumor	Therapy	Nature of the Tissue	Relevant Pathological Features	ER%	PR%	HER2%
B-Met1	Left femoral head	ER+/PR- IDC	Tamoxifen, Arimidex Aromasin, Fulvestrant, Chemotherapy	surgical resection		0	0	0
B-Met2	Right femoral head	ER+/PR- IDC	Letrozole, Fulvestrant	surgical resection		60	0	0
B-Met3	Left iliac bone	ER+/PR- Stage IV IDC	New diagnosis no therapy	surgical resection	PI3KCA mutations in the primary tumour	30	0	0
B-Met4	Right femoral head	ER+/PR- IDC	Tamoxifen, Letrozole Fulvestrant, Chemotherapy	surgical resection		10	0	0
B-Met5	Right femoral head	Triple Negative	Chemotherapy, no HT	surgical resection	p53 mutations, active stromal reaction in the metastatic tissue	50	0	0
B-Met6	Right humerus	ER+/PR+/HER2+ IDC	Letrozole, Herceptin Chemotherapy	biopsy	Her2 amplification in the primary tumour	60	5	3
B-Met7	Left femur	ER+/PR+ IDC	Tamoxifen, Arimidex Aromasin, Fulvestrant, Chemotherapy	surgical resection		100	0	0
B-Met8	Right femur	ER+/PR+ IDC	No therapy (Stage IV)	surgical resection		60	0	0

Supplementary Table 3. Clinical and pathological characterization of bone metastatic tissues obtained from hormonal therapy resistant luminal breast cancer patients and propagated in vitro (HTR-B-Met, MSKCC cohort, Fig. 1).

Supplementary Table 4. Antibodies used in the study

Antibody	Clone	Company	Country	Use
GFP	ab13970	Abcam	USA	IF
ERα	-	Santa Cruz Biotech. Inc	USA	WB
ERα	1D5	Dako, Agilent Technologies	USA	IHC, IF
PgR	1A6	Novocastra Lab	UK	IHC
Ki67	MM1	Novocastra Lab	UK	IHC
IL6	1936	R&D Systems	USA	IHC
CD44	G44-26	BD Pharmingen	USA	IHC, FLOW
CD44	156-3C11	Cell Signaling	USA	IF
CD133	W6B3C1	Miltenyi GmbH	Germany	IHC
CD133	AC133	Miltenyi GmbH	Germany	IF, FLOW
Notch3	M-134	Santa Cruz Biotech. Inc	USA	IHC, IF
Notch3	D11B8	Cell Signaling	USA	WB
Pancytokeratin	wide spectrum clone AE1/AE3	Ventana Medical System Inc	USA	IHC
Mitochondrial Antigen (MAg)	113-1 monoclonal	Biogenex Laboratories	USA	IF
HK-2	C64G5	Cell Signaling	USA	WB
PMK-2	D784A	Cell Signaling	USA	WB
LDHA	N14 sc-27230	Santa Cruz Biotech. Inc	USA	WB
PHDA	C54G1	Cell Signaling	USA	WB
α-Tubulin	10D8	Santa Cruz Biotech. Inc	USA	WB
VDAC1	N18 sc8828	Santa Cruz Biotech. Inc	USA	WB
Stat3	D3Z2G	Cell Signaling	USA	WB
pStat3 (TYR 705)	D3A7	Cell Signaling	USA	WB
pHER2 (pY1248)	2244	Cell Signaling	USA	WB
HER2	06J62	Millipore	USA	WB
pERK1/2	9101	Cell Signaling	USA	WB
ERK1/2	CC-14	Santa Cruz Biotech. Inc	USA	WB
Aromatase	-	Santa Cruz Biotech. Inc	USA	WB

Supplementary Table 4. List of antibodies for immunohistochemistry (IHC), immunofluorescence (IF), flow cytometry (flow) and western blot (WB) analyses.
